# Using emergency department-based inception cohorts to determine genetic characteristics associated with long term patient outcomes after motor vehicle collision: Methodology of the CRASH study  

**DOI:** 10.1186/1471-227X-11-14

**Published:** 2011-09-26

**Authors:** Timothy F Platts-Mills, Lauren Ballina, Andrey V Bortsov, April Soward, Robert A Swor, Jeffrey S Jones, David C Lee, David A Peak, Robert M Domeier, Niels K Rathlev, Phyllis L Hendry, Samuel A McLean

**Affiliations:** 1 Department of Anesthesiology, University of North Carolina, Chapel Hill, North Carolina, USA; 2 Department of Emergency Medicine, University of North Carolina, Chapel Hill, North Carolina, USA; 3 Department of Emergency Medicine, William Beaumont Hospital, Royal Oak, Michigan, USA; 4 Department of Emergency Medicine, Spectrum Health - Butterworth Campus, Grand Rapids, Michigan, USA; 5 Department of Emergency Medicine, North Shore University Hospital, Manhasset, New York, USA; 6 Department of Emergency Medicine, Massachusetts General Hospital, Boston, Massachusetts, USA; 7 Department of Emergency Medicine, St. Joseph Mercy Hospital, Ann Arbor, Michigan, USA; 8 Department of Emergency Medicine, Baystate Medical Center, Springfield, Massachusetts, USA; 9 Department of Emergency Medicine and Pediatrics, University of Florida-Jacksonville, Jacksonville, Florida, USA

## Abstract

**Background:**

Persistent musculoskeletal pain and psychological sequelae following minor motor vehicle collision (MVC) are common problems with a large economic cost. Prospective studies of pain following MVC have demonstrated that demographic characteristics, including female gender and low education level, and psychological characteristics, including high pre-collision anxiety, are independent predictors of persistent pain. These results have contributed to the psychological and social components of a biopsychosocial model of post-MVC pain pathogenesis, but the biological contributors to the model remain poorly defined. Recent experimental studies indicate that genetic variations in adrenergic system function influence the vulnerability to post-traumatic pain, but no studies have examined the contribution of genetic factors to existing predictive models of vulnerability to persistent pain.

**Methods/Design:**

The Project CRASH study is a federally supported, multicenter, prospective study designed to determine whether variations in genes affecting synaptic catecholamine levels and alpha and beta adrenergic receptor function augment social and psychological factors in a predictive model of persistent musculoskeletal pain and posttraumatic stress disorder (PTSD) following minor MVC. The Project CRASH study will assess pain, pain interference and PTSD symptoms at 6 weeks, 6 months, and 1 year in approximately 1,000 patients enrolled from 8 Emergency Departments in four states with no-fault accident laws.

**Discussion:**

The results from this study will provide insights into the pathophysiology of persistent pain and PTSD following MVC and may serve to improve the ability of clinicians and researchers to identify individuals at high risk for adverse outcomes following minor MVC.

## Background

During recent decades, emergency medicine research has continued to expand in scope and increasingly focuses not only on immediate patient outcomes but also on patient outcomes well beyond emergency department (ED) departure. Inception cohort studies examining long term outcomes among patients experiencing acute illness or trauma benefit from enrolling patients in the ED because it is the site of their initial care. In addition, for many patients the ED serves as their only source of care [1,2]. For these reasons, an increasing number of large scale multidisciplinary research projects have utilized ED-based inception cohorts to evaluate long term outcomes after screening, risk stratification, or interventions performed in the ED [3-8].

This report describes the methods of Project CRASH, an example of a type of ED-based inception cohort study which we anticipate will become increasingly common in the future. Project CRASH uses an ED-based inception cohort to enroll patients soon after injury, and examines genetic factors associated with long term patient outcomes. A prior study examined genetic variations in alcohol metabolism as a risk factor for trauma center admission [9], but to our knowledge no studies have used ED-based inception cohorts to examine genetic characteristics associated with long term patient outcomes in the hope of advancing understanding of disease pathophysiology.

The goal of Project CRASH is to gain new insights into the pathophysiology of persistent pain and psychological sequelae after motor vehicle collision (MVC). Approximately 6 million people present to United States EDs each year for care after MVC. More than 90% of these individuals do not have serious physical injury and are discharged to home after ED evaluation [10]. However, persistent post-MVC pain (most commonly neck pain) is experienced by 10-20% of individuals after "minor" MVC [11,12] with significant associated economic cost [13]. The mechanisms by which patients develop acute and chronic pain after minor MVC remain poorly understood [14]. Interestingly, increasing evidence suggests that stress systems, such as the sympathetic nervous system and adrenomedullary hormonal system, may modulate neurosensory processing after trauma exposure, contributing to the development of chronic pain [15]. If a particular physiologic system is involved in the pathogenesis of a disorder, then genetic variations influencing the function of this system would be expected to be associated with vulnerability to develop the disorder and to improve prediction of which individuals will develop this disorder. These concepts are the rationale of the Project CRASH, R01AR056328, "Genetic Predictors of Acute and Chronic Musculoskeletal Pain after Motor Vehicle Collision."

## Methods/Design

### Study Sites

The CRASH study is a prospective multicenter observational cohort study of patients experiencing minor MVC. Study participants are enrolled at research network ED sites and receive initial interview evaluation at the time of the ED visit. Study participant follow-up assessments are performed at 6 weeks, 6 months, and one year. The study research network ("TRYUMPH Research Network") includes Baystate Medical Center, Massachusetts General Hospital, North Shore University Hospital, Shands Jacksonville Hospital, Spectrum Health Butterworth Hospital, St. Joseph Mercy Hospital, and William Beaumont Hospital (2 ED sites). All eight EDs are located in states with "no fault" insurance laws that restrict the right to seek recovery from other parties through the civil-justice system. The study is restricted to states with no-fault accident laws to minimize the likelihood of legal action following MVC, which is a potential confounding factor influencing patient outcome [16]. The data coordinating center for the study is located at the University of North Carolina, Chapel Hill, NC, USA.

### Inclusion Criteria

Patients age 18 to 65 who present to the ED within 24 hours after minor MVC and who are unlikely to require admission are screened for eligibility. Patients with injuries likely to require hospitalization, fractures (other than small bone fractures), major lacerations (defined as a laceration more than 20 cm in length or more than four lacerations requiring sutures), intracranial injury, or spinal injury (defined as vertebral fracture or dislocation, or new neurologic deficit) are excluded. Patients who are deemed eligible but subsequently admitted overnight to the hospital are excluded. Patients who go to an ED observation area for a brief period (e.g. "6 hour rule out") remain eligible. Patients are excluded if they are prisoners, pregnant, not alert and oriented, or unable to read and understand English. Patients are also excluded if they take β-receptor antagonist or if they take opioids on a daily basis above a total daily dose of 20 mg of oxycodone or equivalent. In addition, due to the effects of ethnicity on genetic risk factor assessment (population stratification bias [17] that may require different ethnicities to be analyzed separately) and budget restrictions limiting sample size, enrollment is limited to European Americans. After assessment for eligibility, signed informed consent is obtained from all patients enrolled in the study.

### Patient screening and consent

Patient screening and assessment are performed using web-based study assessment forms. Research assistants (RAs) complete a web-based patient screening questionnaire for each patient presenting to the ED for evaluation after MVC during day and evening hours when study site research team members are staffing the ED. The screening form prompts the RA to complete a series of questions. If participants are eligible for participation based on screening questionnaire responses, the RA is automatically advanced to the ED assessment interview web survey. If participants are not eligible, the reason for ineligibility is stored by the system. If patients are eligible, they are offered participation in the study. Signed informed consent is obtained from willing participants.

### Blood collection for DNA

After consent is obtained, a single blood sample (8.5 cc) is collected using a PAXgene DNA storage tube (http://www.preanalytix.com). When possible, this blood sample is obtained when blood is collected as part of the patient's medical evaluation, to avoid additional phlebotomy. Each blood specimen is labeled with a barcode sticker, which serves as a unique identifier for the sample. After the barcode sticker is placed on the sample, the barcode is scanned using a reader wand which enters the barcode number into a web-based tracking system and links the number with the participant's identification number. The barcode is also scanned at the time of shipment from the study site to the genotyping facility, and at the time of receipt by the genotyping facility, to maintain blood sample chain of custody. PAXgene DNA storage tubes are stored at 4°C (standard refrigeration) for up to two weeks [18] at the study site prior to batch shipment to Cogenics, Inc., (Morrisville, NC).

### ED Interview

ED assessments are conducted by trained research assistants using a standardized web-based questionnaire on laptop computer. Back-up paper copies are used by RAs if hospital wireless internet service is unavailable. The ED interview begins with the collection of patient contact information, including information on two potential alternative contacts. Subsequent interview assessments include the collection of detailed information regarding the collision event, current somatic and psychological symptoms, past somatic and psychological symptoms, and general health and medication use (Table 1; Additional Files 1 and 2). Participants are compensated $80 for completing the ED evaluation.

**Table 1:** Study question domains, specific measures, and times of assessment.

Domain	Measure	ED	1 M	6 M	1 Y*
MVC injury events	Questionnaire	**●**			
Current pain symptoms	NRS**, Regional Pain Scale	**●**	**●**	**●**	**●**
	Scale[25]				
Optimism	Life Orientation Test-Revised[26]	**●**			
Current whiplash symptoms	Quebec classification[11]	**●**			
Current somatic symptoms	Standardized questionnaire	**●**	**●**	**●**	**●**
Distress after MVC	Peritraumatic Distress Inventory[27]	**●**			
Dissociative symptoms after MVC	Critical Events Perception Inventory[28]	**●**			
Catastrophizing	Pain Catastrophizing scale[29]	**●**			
Current somatic symptoms	Somatic symptom interview[30]				
Pre-MVC General health	SF-12[31]	**●**			
Pre-MVC Anxiety symptoms	STPI^¥ ^Form Y[32]	**●**			
Pre-MVC Depressive symptoms	CES-D°[33]	**●**			
PTSD^† ^symptoms	Impact of Event Scale-Revised[34]		**●**	**●**	**●**
General health	SF-12[31]		**●**	**●**	**●**
Demographic information	Standard items	**●**		**●**	**●**
Medication Use	Standard items	**●**	**●**	**●**	**●**
Alcohol and drug use	TWEAK[35], Substance Abuse Outcomes Module[36]	**●**		**●**	**●**
New injury or re-injury	Standard Items		**●**	**●**	**●**
Pain interference	Brief Pain Inventory (pain interference questions)[37]		**●**	**●**	**●**
Service utilization, disability/litigation claims	Standard items		**●**	**●**	**●**

*ED = Emergency Department, 1 M = 1 month, 6 M = 6 months, 1 Y = w year, **NRS = Numeric rating scale, ^¥^State Trait Personality Inventory, ^†^PTSD = Posttraumatic Stress Disorder, °Center for Epidemiologic Studies Depression Scale

### Data Extraction

During the week following the completion of the ED interview, study site RAs extract data from the participant's medical record using a standardized web-based data extraction form. Fields on this data extraction form provide explicit definitions of all variables. Information collected includes chief complaint, vital signs, past medical history, medications given in the ED and prescribed at discharge, injuries described in the medical record, and results of radiologic studies performed in the ED. All aspect of the emergency medical record including physician notes, nursing notes, and physician orders were used for completion of data extraction. Injury information was used to generate Abbreviated Injury Severity Scores [19].

### Follow-Up Assessments

Follow-up assessments are completed at 6 weeks, 6 months, and 1 year post-enrollment with assessment made during a 4 week window for each follow-up time point (Additional File 3). Information collected at each follow-up is identical, except that drug and alcohol use is reassessed at only the 6 month and 1 year time points. Participants are able to complete the interview online, by telephone, or by mail. Information packets are sent that remind participants that it is time to complete their next assessment. These packets include the actual questionnaires to be completed during the interview; if participants choose to complete their assessment via mail they can do so or they can follow along with the questionnaires at home during a telephone interview. Follow up interviews are scheduled at a time (day or evening) that is convenient for the participant. Three and nine months after enrollment, participants also complete a contact information update via mail. Assessments collect detailed information regarding re-injury or new injury events, as well as information regarding involvement in a litigation or disability claim, missed work or other activities, somatic symptoms (including pain symptoms), psychological symptoms, and functional interference due to pain (Table 1). For any body region for which pain is reported, the patient is also asked whether the pain is due to (began after) the MVC. Health care utilization assessments are also evaluated and include: medication use; visits to the ED, primary physician, specialist physicians, or alternative medicine providers; and hospitalization. Participants are compensated $50, $60, and $70 after completing the 6 week, 6 month, and 1 year follow-up interviews, respectively.

### Protection of Patient Privacy

A number of methods are used to maintain the confidentiality of study data. A Certificate of Confidentiality has been obtained from the National Institutes of Health to protect identifiable research information from forced disclosure [20]. All research assistants involved in the study complete training in the protection of patient confidentiality through the Collaborative Institutional Training Initiative [21]. Signed consent is obtained from all patients to perform all assessments and to allow future analysis of de-identified blood specimens. A unique patient identifier is used to link patient identifying information to results of questionnaires and genetic information, and all analyses are conducted on de-identified data. The study has been approved by the institutional review boards of all participating institutions.

### Sample Size Estimate

Sample size was calculated to determine the number of patients needed to assess the influence of selected adrenergic system-related genes on the presence of persistent moderate or severe neck pain with adequate power. Because the genes to be assessed contain approximately 59 haplotypes, a Bonferroni corrected alpha was set at. 00085 (.05/59). Estimates of the number of patients needed to identify the effect sizes obtained for candidate haplotypes examined in our pilot data ranged from 171 to 1,295 (alpha = .00085, beta = .8). These estimates were believed to be conservative because 1) the inclusion of non-genetic factors should reduce the unexplained variance in the model and increase power to detect the influence of genetic factors, 2) analyses will utilize repeated-measures logistic regression, which will increase power, and 3) for some genes, only specific risk haplotypes will be assessed so the actual number of haplotypes will likely be less than 59. Based on these analyses, recruitment of 936 patients is planned, in order to achieve at least 795 patients completing follow-up time points (estimated follow-up rate of 85%).

### Data Analysis

DNA is extracted (average PAXgene DNA yield 150 μg to 500 μg) and targeted genotyping of single nucleotide polymorphisms (SNPs) is performed using the Sequenom (Sequenom, Inc., San Diego, CA) platform. When possible, a haplotype-based approach to genetic analyses is utilized, because previous studies suggest that a haplotype-based approach to genetic analyses is often most useful. This is because the overall functional state of a gene may not be easily deduced from information regarding a single SNP [22]. For example, haplotype-dependent secondary RNA structure can have a much greater influence on function than a functional SNP within this haplotype [22]. To construct haplotypes, both functional SNPs previously shown to affect gene function and also tag SNP markers within each gene locus (to capture haplotypic diversity) are genotyped. Two hapmap samples, for which the entire genome sequence is known, and 2 repeat samples are included in each genotyping batch to assess genotypic accuracy and reliability. Haplotypes are then constructed for each of the genetic risk factors assessed using the Haploview software program. Polymorphisms of interest include genetic variations influencing catecholamine levels (monoamine oxidase A, monoamine oxidase B, norepinephrine transporter, catechol-O-methyltransferase) and adrenoreceptor function (α1A, α1B, α1D, α2A, α2B, α2C, β1, β1). Quality control of genetic data includes assessment of call rates for each SNP, identification of samples with call rates < 90%, and test of Hardy-Weinberg equilibriums for each locus. Genetic data results are then merged with phenotypic data and analyzed using standard statistical methods. Primary and secondary analyses evaluate genotypic and phenotypic predictors of persistent pain and psychological sequelae.

## Discussion

ED-based inception cohort studies such as Project CRASH, which combine genotypic and phenotypic data collection, are likely to become increasingly common for several reasons. First, as the cost of genotyping continues to decline, and as our knowledge of genetic polymorphisms influencing physiologic processes continues to increase, the value (cost effectiveness) of these studies will continue to grow. Second, genetic (and epigenetic) techniques represent one useful method to address a critical shortfall in current medical knowledge: our ability to identify those at high risk for a particular disease outcome has rapidly outpaced our knowledge of the mechanisms mediating the outcome. That is, increasingly we can identify "who", but we do not know "why". Including genetic factors in traditional epidemiologic studies enhances the ability of study findings to yield clues regarding disease pathogenesis, by determining the physiologic systems or pathways contributing to disease outcomes, and by determining the environmental conditions under which these outcomes occur (gene × environment interactions). Further knowledge of the physiologic systems which mediate disease outcomes has the potential to enhance our ability to identify high-risk individuals and/or to suggest novel preventive interventions. These studies require relatively large number of patients, which ED-based networks can provide.

To most rapidly bring the benefits of these types of studies to our patients, organizations within academic emergency medicine should continue to look for opportunities to bring knowledge of genetic epidemiologic methods to emergency medicine researchers. The above description of Project CRASH is hopefully useful in this regard. Fortunately, the genetics field continues to grow exponentially, with an increasing array of opportunities to rapidly get "up to speed" regarding performing and interpreting genetics studies. For example, there are now a number of societies focused on genetic studies in humans (e.g. Society for Human Genetics), and online genetic education resources are available through the National Human Genome Research Institute (http://www.genome.gov/10000464), the Human Genome Project (http://www.ornl.gov/sci/techresources/Human_Genome/posters/chromosome/), and other educational organizations.

### Difficulties and Potential Limitations

A current limitation of epidemiologic studies examining genetic factors is that they often must be performed within a particular ethnicity. As described above, Project CRASH is limited to European Americans only. This is because the prevalence of specific genetic variations often varies between ethnicities. For example, haplotype frequencies for COMT genes are different between Caucasians, African Americans, and Asians [23]. This necessitates stratified analyses, and the size of each strata must be large, often limiting a study to a single strata within the context of the budget of a particular grant mechanism.

Several methods are now available to address this problem of population stratification. One of them is principal components analysis of a set of ancestry-informative markers (i.e. SNPs which have different frequencies among populations from different regions) to identify population ancestral heterogeneity [24]. However, the accuracy of these methods vs. stratified analyses remains a matter of debate. As with other areas of genetic research, the only constant is that techniques and methods will continue to rapidly change.

## Competing interests

The authors declare that they have no competing interests.

## Authors' contributions

SM developed the study concept and design. LB, AS, RS, JJ, DL, DP, RD, NR, and PH contributed to the acquisition of data and supervised the study. AB provided statistical expertise and assisted in study design. TPM and SM drafted the manuscript, and all authors made critical revisions of the manuscript for important intellectual content. TPM and SM take responsibility for the paper as a whole. All authors read and approved the final manuscript.

## Pre-publication history

The pre-publication history for this paper can be accessed here:

http://www.biomedcentral.com/1471-227X/11/14/prepub

## Supplementary Material

Additional file 1 **CRASH Methods ED Assessment Part 1**. Part 1 of the 2 part survey administered in the Emergency Department. Click here for file

Additional file 2 **CRASH Methods ED Assessment Part 2**. Part 2 of the 2 part survey administered in the Emergency Department. Click here for file

Additional file 3 **CRASH Methods Follow-Up Questionnaire**. The survey administered at each follow-up interval. Click here for file
