# Supplementary material for: Using emergency department-based inception cohorts to determine genetic characteristics associated with long term patient outcomes after motor vehicle collision: Methodology of the CRASH study
Source: BMC Emerg Med. 2011 Sep 26;11:14. doi: 10.1186/1471-227X-11-14 (PMC3188467; doi:10.1186/1471-227X-11-14)
Supplement: Additional file 1 — CRASH Methods ED Assessment Part 1. Part 1 of the 2 part survey administered in the Emergency Department. [file 1471-227X-11-14-S1.DOC]

**INITIAL CONTACT INFORMATION**

**(*Text is read aloud to patient. Information in italics is for RA only.)***

Your responses to all of these questions are entirely confidential. The confidentiality of the information you give me is protected by federal law.

First I would like to collect some information that will help us to reach you when it’s time for your follow-up interviews. This information will only be used to locate you for your follow-up interviews and will not be shared with anyone else outside the Project CRASH Team. Your contact information will be destroyed at the conclusion of the study.

1. What is your current address?

Street: ___________________________________________________________

City: _____________________________ State:_______ Zip:________________

1. Do you plan on moving or changing your primary address in the next two months?

____ No *(GO TO 5)*

____ Yes *(GO TO 3)*

1. On what date will you be moving? If you are not certain, please give me your best guess.

___/___/____

1. What will be your new street address?

Street:_______________________________________________________

City:_____________________________ State:_______ Zip:_____________

□ Unknown *(Check Box*

1. Now I am going to ask you about phone numbers where we can reach you. For each phone number, I will also ask you when the best times are to try to reach you at that number.

|  |  | **Best times to reach you at that number:** | | | ***Skip these questions if the participant will not be moving in the next two months.*** | |
| --- | --- | --- | --- | --- | --- | --- |
| TYPE | PHONE | Day of the week | Start Time | End Time | Will your upcoming move change this number? | *If yes,* if you know, what will your new number be? |
| Home | XXX-XXX-XXXX |  | _ _ :_ _ | _ _ :_ _ | Yes / No | XXX-XXX-XXXX |
|  |  |  | _ _ :_ _ | _ _ :_ _ |  |  |
|  |  |  | _ _ :_ _ | _ _ :_ _ |  |  |
| Cell | XXX-XXX-XXXX |  | _ _ :_ _ | _ _ :_ _ | Yes / No | XXX-XXX-XXXX |
|  |  |  | _ _ :_ _ | _ _ :_ _ |  |  |
|  |  |  | _ _ :_ _ | _ _ :_ _ |  |  |
| Work/Other | XXX-XXX-XXXX |  | _ _ :_ _ | _ _ :_ _ | Yes / No | XXX-XXX-XXXX |
|  |  |  | _ _ :_ _ | _ _ :_ _ |  |  |
|  |  |  | _ _ :_ _ | _ _ :_ _ |  |  |

1. Which is the best number for us to try first to reach you? (Check one)

Home ___

Cell ___

Work or Other ___

1. It would be helpful if you would provide us with the name and address of two people who could give us your new contact information if you move. These should be people who do not live with you. We would contact these people only if we are unable to reach you using the other information that you have given us.

1. Do you have access to the internet?

____No

____Yes (GO TO 8A)

a. Do you use email regularly?

____ No

____ Yes

b. ***If yes****:* Would it be OK for us to contact you by email? We would only send emails to you that are related to your participation in this study.

____ No

____ Yes

**c. *If yes***: Please tell me any email address(s) that you check regularly:

#1 ___________________________________________________

#2 ___________________________________________________

#3 ___________________________________________________

1. When it is time for your follow-up assessments, one of our CRASH research team members will contact you to set up a time to talk to you by phone. We will mail you all the questions we are going to ask you before we call, to help the interview go quickly.

If you would like, we can also provide you with information about a secure website where you can log on and complete your interview. If you complete the interview on the internet, you will not need to complete a telephone interview. You will receive the same payment for completing the interview questions, regardless of whether you answer the questions using the internet or by telephone interview. The questions are exactly the same either way.

Would you like the option to complete your interview using the internet?

_____No ____ Yes

**Injury Events Questionnaire**

Next, I would like to ask you some questions about the accident. For these questions, “vehicle” refers to the motor vehicle you were in, whether it was a car, motorcycle, bus, pickup truck, or other vehicle.

1. About what time of day or night did the accident happen?

*(try to get to nearest ½ hour – format 24 hour time)*

__ __: __ __

2. What **type of vehicle** were **you** in when the accident happened? Please select one option.

**1** ____ Passenger Car

**2** ____ Pick-up truck

**3** ____ Sports Utility Vehicle (SUV)

**4** ____ Full-size van

**5** ____ Mini-van

**6** ____ Full size truck

**7** ____ Bus

**8** ____ Motorcycle

**9 ____** Other *(please describe)* ________________________

2a. If you were on a **motorcycle** where were you seated?

**1** Driver

**2** Motorcycle Passenger

**3____** Other *(please describe)* ________________________

3. Where were **you** **sitting** in the vehicle when the accident happened? Please select one option.

*(Have patient respond, then ask follow-up question if necessary to identify correct response.)*

**1** ____ Driver

**2** ____ Center-front passenger

**3** ____ Right-front passenger

**4** ____ Left-second seat passenger

**5** ____ Center-second seat passenger

**6** ____ Right-second seat passenger

**7** ____ Left-third seat passenger

**8** ____ Center-third seat passenger

**9** ____ Right-third seat passenger

**10** ____ Passenger in bus or large van

**11** ____ Other *(please describe)* ________________________

4. What was the speed limit of the road you were traveling along?

(*If patient doesn’t know, ask for best guess)*

   miles per hour

5. About **how fast** do you think the vehicle **you** were in was moving when the accident occurred?

**1** ____ The vehicle was moving backwards

**2** ____ Vehicle was stopped

**3** ____ 1 - 10 mph

**4** ____ 11 - 20 mph

**5** ____ 21 – 30 mph

**6** ____ 31 – 40 mph

**7** ____ 41 – 50 mph

**8** ____ 51 – 60 mph

**9** ____ 61 – 70 mph

**10** ____ 71 – 80 mph

**11** ____ 81 – 90 mph

**12** ____ 91 – 100 mph

**13** ____ > 100 mph

6. Did the vehicle you were in contact another vehicles or objects during the accident? (select all that apply) *(patient will describe, RA classify as below)*

1 ____ Another passenger vehicle

**2** ____A Motorcycle

**3**____ A bus

**4** ____A large truck

**5** ____ A sign post

**6** ____ A telephone pole or tree

**7** ____ A bridge abutment or median barrier

**8** ____ Other object *(please describe)* ________________________

**9**_____Nothing (spinout)

7. Please describe the location(s) of the main or primary damage to **your** vehicle because of the accident: (select all that apply) *(RA to ask patient for clarification of damage location if necessary)*

**1** ____ No damage

**2** ____ Front

**3** ____ Rear end

**4** ____ Left side, forward of doors

**5** ____ Left side, at doors

**6** ____ Left side, to rear of doors

**7** ____ Right side, forward of doors

**8** ____ Right side, at doors

**9** ____ Right side, to rear of doors

**10** ____ Top/roof

**11** ____ Undercarriage

**12** ____ Other (please describe)___________________________________

**13** ____ I don’t know

8. Which of the following phrases best describes the **extent** of damage, overall, to the vehicle you were traveling in? (*If patient doesn’t know, ask for best guess)*

**1** ____ There was no damage

**2** ____ Damage was minor

**3** ____ Damage was moderate

**4** ____ Damage was severe, car was not drivable

**If vehicle impacted with another vehicle, answer question #9, otherwise skip to #10:**

9. How fast do you think the **other vehicle was moving** when it impacted with your vehicle?

*(Ask for their best guess if they are not sure)*

**1** ____ It was stopped

**2** ____ Slowly at less than 15 mph

**3** ____ At a moderate speed of 15 to 35 mph

**4** ____ Faster than 35 mph but less than 55 mph

**5** ____ More than 55 mph

**6** ____ I don’t know

10. Were you using a seatbelt at the time of the accident?

**0** ____ No **1** ____ Yes

10a. **If yes**, were you using a lap belt, a shoulder belt, or both?

**1** ____ Combination lap and shoulder belt

**2** ____ Shoulder belt only (*e.g., automatic shoulder belt*)

**3 ____** Lap belt only *(e.g., put shoulder belt behind back)*

11. Did any airbags in your vehicle go off (deploy) during the accident?

**0** ____ No **1** ____ Yes **2**____I don’t know

11a. **If yes**, where were the airbags located in the car that went off? (select all that apply)

*(Patient describes and RA fills in, prompting patient if necessary)*

**1** ____ Airbag in steering wheel

**2** ____ Airbag in dashboard on passenger side

**3** ____ Airbag in driver seat

**4** ____ Airbag in driver door

**5** ____ Airbag in passenger seat

**6** ____ Airbag in passenger door

**7** ____ Airbag in roof side rail *(i.e., above door)*

12. Did any part of your body hit or make contact with anything in your vehicle?

**0** ____ No **1** ____ Yes **2**____I don’t know

12a. **If yes**, what part(s) of your body made contact with the vehicle? (select all that apply)

**1** ____ Head/face **6** ____ Thighs

**2** ____ Shoulders **7** ____ Knees

**3** ____ Arms/hands **8** ____ Legs

**4** ____ Chest/back **9** ____ Ankles/feet

**5** ____ Hips/pelvis

13. Did you lose consciousness during the accident? That is, were you knocked out during the accident?

**0** ____ No **1** ____ Yes

13a. **If yes**, for how long were you knocked out?

**1** ____ Just a few seconds (select)

**2** ____ Less than a minute (select)

**3** ____ One minute or more

13b. **If more than one minute**, what is your best guess about how long you were knocked out/unconscious?

  (*record in minutes of time*)

14. Do you have amnesia for some of the events of the accident? That is, are there parts of the accident that you don’t remember?

**0** ____ No **1** ____ Yes

14a. **If yes**, how much of the accident do you think you don’t remember?

**1** ____ Just a few seconds

**2** ____ Less than a minute

**3** ____ One minute or more

14b. **If more than one minute**, what is your best guess of the period of time that you don’t remember?

  (*record in minutes of time*)

15. How life threatening was your motor vehicle collision? Please rate how close you came to dying in the collision on a 0 to 10 scale, where zero means that your life was not threatened at all, and 10 means that you came very close to being killed, or could easily have been killed.

________ (Record 0-10 response)

16. Did you come to the emergency department directly from the scene of the accident?

**0** ____ No **1** ____ Yes

16a. **If yes**, how were you transported to the hospital? Please select one option.

**1** ____ Ambulance

**2** ____ I drove myself

**3** ____ A friend or family member drove me

**4** ____ Other *(please describe)* ________________________

17. If you were transported to the hospital by ambulance, were you strapped to a board by the paramedics?

**0** ____ No **1** ____ Yes

18. Was anyone else in the car **with you** at the time of the accident?

**0** ____ No **1** ____ Yes

18a. **If yes**, how many people were in the vehicle?

 passengers

18b. **If yes**, how badly was each passenger injured? *(RA should lead participant through the following chart recording an injury category for each passenger using the severity key below. If there were more than six passengers in the vehicle then record categories for the six most severely injured passengers)*

|  | **Severity of Injury (use severity key)** |  |  | **Severity Key** |
| --- | --- | --- | --- | --- |
| **Passenger 1** |  |  |  | 0 = No Injury |
| **Passenger 2** |  |  |  | 1 = Minor Injury |
| **Passenger 3** |  |  |  | 2 = Moderate Injury |
| **Passenger 4** |  |  |  | 3 = Severe Injury |
| **Passenger 5** |  |  |  | 4 = Deceased |
| **Passenger 6** |  |  |  | 5= Don't Know or Refused |

19. **If another vehicle was involved**: Do you know if anyone was injured in the **other vehicle**?

**0** ____ No **1** ____ Yes **2**____I don’t know

19a. **If yes**, what type of injury did the most severely injured passenger in the other vehicle sustain? (*RA categorizes based on their best estimate)*

**1** ____ Minor Injury

**2** ____ Moderate Injury

**3** ____ Severe injury

**4** ____ Deceased

**5 ____** Don’t Know

**Post-MVC Quebec Classification Questions**

Now I would like to ask you some questions about any pain that you are having.

1. When I press on your neck, does it feel tender or sore where I press? *(examiner palpates over the trapezius and along both sides of the lower neck)*

**0** ____ No

**1** ____ Yes

**2** ____ Declined exam

1a. **If yes**, how bad is it right now on a scale of zero to ten, where zero means no tenderness or soreness, and ten means tenderness or soreness as severe as it could possibly be?

*Record 0-10 response*  .  *(allow and record “.5” responses)*

2. Is your ability to move your neck limited by pain, stiffness, or soreness? *(ask them to slowly tilt their head toward each shoulder)*

**0** ____ No

**1** ____ Yes

2a. **If yes**, on a scale of zero to ten, where zero means no limitation in neck movement, and ten means you can’t move your neck at all because of pain, stiffness, and soreness, how bad is it right now?

*Record 0-10 response*  .  *(allow and record “.5” responses)*

3. Do you have pain or numbness radiating from your neck to your shoulder, arm, or other part of the body, or do your neck or arms feel numb or have decreased sensation?

**0** ____ No

**1** ____ Yes

**Post-MVC** **Pain Symptoms Question**

4. Are you having any pain or discomfort in any part of your body **right now**? (*Skip this question if the patient has already reported spontaneous pain in answering the above questions).*

**0** ____ No

**1** ____ Yes

*🡪****If no****, skip the next section containing the Regional Pain Scale*

**Post-MVC Regional Pain Scale**

Now, I am going to ask you about the amount of pain and/or tenderness that you are feeling right now in a number of different body areas. For each body area, please chose the number that best describes your pain or tenderness on a scale from 0-10, where 0 is no pain or tenderness and 10 is pain or tenderness as severe as it could be. (*For each body area where patient is having pain, ask “Is this pain related to your motor vehicle accident?” If pain is obviously related to accident (e.g. laceration in area), RA may record response without asking patient.)*

Is this pain related to your motor vehicle accident?

**NO SEVERE**

**PAIN PAIN**

1. Head 0 1     2     3     4     5     6     7     8     9    10 ____NO ____YES

2. Neck 0 1     2     3     4     5     6     7     8     9    10 ____NO ____YES

3. Left Jaw 0 1     2     3     4     5     6     7     8     9    10 ____NO ____YES

4. Right Jaw 0 1     2     3     4     5     6     7     8     9    10 ____NO ____YES

5. Left Shoulder 0 1     2     3     4     5     6     7     8     9    10 ____NO ____YES

6. Right Shoulder 0 1     2     3     4     5     6     7     8     9    10 ____NO ____YES

7. Left Upper Arms 0 1     2     3     4     5     6     7     8     9    10 ____NO ____YES

8. Right Upper Arms 0 1     2     3     4     5     6     7     8     9    10 ____NO ____YES

9. Left Lower Arms 0 1     2     3     4     5     6     7     8     9    10 ____NO ____YES

10. Right Lower Arms 0 1     2     3     4     5     6     7     8     9    10 ____NO ____YES

11. Chest 0 1     2     3     4     5     6     7     8     9    10 ____NO ____YES

12. Upper Back 0 1     2     3     4     5     6     7     8     9    10 ____NO ____YES

13. Lower Back 0 1     2     3     4     5     6     7     8     9    10 ____NO ____YES

14. Abdomen 0 1     2     3     4     5     6     7     8     9    10 ____NO ____YES

15. Left Hip 0 1     2     3     4     5     6     7     8     9    10 ____NO ____YES

16. Right Hip 0 1     2     3     4     5     6     7     8     9    10 ____NO ____YES

17. Left Upper Leg 0 1     2     3     4     5     6     7     8     9    10 ____NO ____YES

18. Right Upper Leg 0 1     2     3     4     5     6     7     8     9    10 ____NO ____YES

19. Left Lower Leg 0 1     2     3     4     5     6     7     8     9    10 ____NO ____YES

20. Right Lower Leg 0 1     2     3     4     5     6     7     8     9    10 ____NO ____YES

21. On a scale of zero to ten, where zero means no pain and ten equals pain as severe as it could possibly be, what is the **intensity** of your pain **right now,** considering any or all of your pains together?

***Record 0-10 response ________ (allow and record “.5” responses)***

**Post-MVC Somatic Symptoms**

Now, I am going to ask you about other symptoms that some people have after a motor vehicle collision. Please rate how much of a problem you had or are having after the accident, where zero means no problem and 10 means a major problem.

**NO MAJOR**

**PROBLEM PROBLEM**

1. Headache 0 1         2          3          4          5          6         7         8          9         10

2. Dizziness 0 1         2          3          4          5          6         7         8          9         10

3. Nausea 0 1         2          3          4          5          6         7         8          9         10

4. Noise Sensitivity 0 1         2          3          4          5          6         7         8          9         10

5. Light Sensitivity 0 1         2          3          4          5          6         7         8          9         10

6. Concentration Difficulty 0 1         2          3          4          5          6         7         8          9         10

7. Fatigue 0 1         2          3          4          5          6         7         8          9         10

8. Taking longer to think 0 1         2          3          4          5          6         7         8          9         10

9. Blurred Vision 0 1         2          3          4          5          6         7         8          9         10

10. Double Vision 0 1         2          3          4          5          6         7         8          9         10

11. Restlessness 0 1         2          3          4          5          6         7         8          9         10

Now, I am going to ask you about any problems you have had in the past, before your accident today, with pain or aching in any part of your body:

**Pre-MVC Pain Symptoms Question**

12. During (*previous named month)*, did you have any problems with pain, tenderness, or discomfort in any part of your body?

**0** ____ No

**1** ____ Yes

***If no****, skip the next section containing the Regional Pain Scale****.***

**Pre-MVC Regional Pain Scale**

Next, we would like to know where you had pain and/or tenderness in (*previously named month)*. I am going to ask you about the amount of pain and/or tenderness you had in (*previously named month)* in a number of different body areas. For each body area please chose a number that best describes your pain and/or tenderness on a scale from 0-10, where 0 is no pain or tenderness and 10 is pain or tenderness as severe as it could be.

**NO SEVERE**

**PAIN PAIN**

1. Head 0 1      2      3      4      5      6       7      8      9    10

2. Neck 0 1      2      3      4      5      6       7      8      9    10

3. Left Jaw 0 1      2      3      4      5      6       7      8      9    10

4. Right Jaw 0 1      2      3      4      5      6       7      8      9    10

5. Left Shoulder 0 1      2      3      4      5      6       7      8      9    10

6. Right Shoulder 0 1      2      3      4      5      6       7      8      9    10

7. Left Upper Arms 0 1      2      3      4      5      6       7      8      9    10

8. Right Upper Arms 0 1      2      3      4      5      6       7      8      9    10

9. Left Lower Arms 0 1      2      3      4      5      6       7      8      9    10

10. Right Lower Arms 0 1      2      3      4      5      6       7      8      9    10

11. Chest 0 1      2      3      4      5      6       7      8      9    10

12. Upper Back 0 1      2      3      4      5      6       7      8      9    10

13. Lower Back 0 1      2      3      4      5      6       7      8      9    10

14. Abdomen 0 1      2      3      4      5      6       7      8      9    10

15. Left Hip 0 1      2      3      4      5      6       7      8      9    10

16. Right Hip 0 1      2      3      4      5      6       7      8      9    10

17. Left Upper Leg 0 1      2      3      4      5      6       7      8      9    10

18. Right Upper Leg 0 1      2      3      4      5      6       7      8      9    10

19. Left Lower Leg 0 1      2      3      4      5      6       7      8      9    10

20. Right Lower Leg 0 1      2      3      4      5      6       7      8      9    10

21. On a scale of zero to ten, where zero means no pain and ten equals pain as severe as it could possibly be, how would you rate your **overall pain intensity** during (previous named month)?

*Record 0-10 response*  .  *(allow and record “.5” responses)*

22. If no **neck pain** reported in past month on Regional Pain Scale (*if reported neck pain, skip*).

**neck pain**

Have you ever had problems with in the past?

**0** ____ No **1** ____ Yes

23. If no **back pain** reported in past month on Regional Pain Scale (*if reported back pain, skip*).

Have you ever had problems with **back pain** in the past?

**0** ____ No **1** ____ Yes

**Pre-MVC Somatic Symptoms**

Now I am going to ask you about any problems you may have had with other symptoms that some people commonly have. Please rate how much of a problem you had with the following symptoms in (*previous named month)*, where zero means no problem, and 10 means a major problem.

**NO MAJOR**

**PROBLEM PROBLEM**

1. Headaches 0 1         2          3          4          5          6         7         8          9         10

2. Dizziness 0 1         2          3          4          5          6 7         8          9         10

3. Nausea 0 1         2          3          4          5          6         7         8          9         10

4. Noise Sensitivity 0 1         2          3          4          5          6         7         8          9         10

5. Light Sensitivity 0 1         2          3          4          5          6         7         8 9 10

6. Concentration Difficulty 0 1         2          3          4          5          6         7         8          9         10

7. Taking longer to think 0 1         2          3          4          5          6         7         8          9         10

8. Blurred Vision 0 1         2          3          4          5          6         7         8          9         10

9. Double Vision 0 1         2          3          4          5          6         7         8          9         10

10. Restlessness 0 1         2          3          4          5          6         7         8          9         10

11. Upset Stomach 0 1         2          3          4          5          6         7         8          9         10

12. Persistent Fatigue 0 1         2          3          4          5          6         7         8          9         10

13. Sensitive or tender skin 0 1         2          3          4          5          6         7         8          9         10

14. Ringing in ears 0 1         2          3          4          5          6         7         8          9         10

15. Itchy eyes or skin 0 1         2          3          4          5          6         7         8          9         10

16. Racing heart 0 1         2          3          4          5          6         7         8          9         10

17. Insomnia or difficulty 0 1         2          3          4          5          6         7         8          9         10

sleeping

18. Hands trembling or shaking 0 1         2          3          4          5          6         7         8          9         10

19. Feeling faint 0 1         2          3          4          5          6         7         8          9         10

20. Abdominal pain 0 1         2          3          4          5          6         7         8          9         10

21. Constipation and/or 0 1         2          3          4          5          6         7         8          9         10

diarrhea

**Time to Recovery Questions**

The next few questions are about your recovery from your motor vehicle collision.

1. How long do you think it takes for a person to **physically** recover from the kind of accident or injury you just had? What is your best guess right now about how long it will take?

 ***(Convert participant answer into days)***

***1 week = 7 days***

***1 month = 30 days***

***1 year = 360 days***

2. How long do you think it takes for a person to **emotionally** recover from the kind of accident or injury you just had? What is your best guess right now about how long it will take?

 ***(Convert participant answer into days)***

***1 week = 7 days***

***1 month = 30 days***

***1 year = 360 days***

**Certainty of Recovery Question**

*3. Ask the subject:* How certain, or sure, are you that you will fully recover from this accident, if on a scale of 0 to 10, the 0 is “certain that you will not recover” and the 10 is “certain that you will fully recover?

0 1 2 3 4 5 6 7 8 9 10

I am certain that I am certain that

I **will not** recover I **will fully recover**

**Pre-MVC Medications**

Now I’m going to ask some questions about any medications you may take.

1. In the **past month**, have you taken any over-the-counter pain medicines, such as Tylenol, Motrin, or Advil?

**0** ____ No

**1** ____ Yes

2. In the **past month**, did you take any prescription pain medicines? (Vicodin, Darvocet, or Tylenol #3, etc.)

**0** ____ No

**1** ____ Yes

Now I would like to make a list of all the medications that you currently take including the pain medications we just talked about. Please tell me all the medications you take even if you just take them once in a while. Include medicines that you take “over the counter” as well as medications that the doctor prescribes for you. (*RA may use previous information collected in ED and patient information to fill out medication information.)*

*For each medication ask, “Do you take this on a regular basis, or do you just take this as needed?”, and then fill in the corresponding chart below.*

Route and Units Guide

| Routes | | Applicable Units |
| --- | --- | --- |
| Oral/PO | Liquids | micrograms (mcg) |
|  |  | milligrams (mg) |
|  |  | grams (g) |
|  |  | grains |
|  |  | teaspoons (tsp) |
|  |  | tablespoons (tbsp) |
|  |  | milliliters (ml) |
|  | Pills | micrograms (mcg) |
|  |  | milligrams (mg) |
|  |  | grams (g) |
|  |  | grains |
|  | Sub-lingual | sprays |
|  |  | tablets |
|  |  | milligrams |
| Injections (IV, Subcutaneous, IM) | | units |
|  |  | micrograms (mcg) |
|  |  | milligrams (mg) |
|  |  | ampules (amps) |
|  |  | milliliters (ml) |
| Inhaler | | puffs |
| Transcutaneous/Patch/Topical | | patches |
|  |  | vials |
|  |  | teaspoons (tsp) |
|  |  | inches |
| Eye/Ear Drop/Ointment | | drops (ggt) |
| Rectal | | suppository |
|  |  | tablet |
| Nasal/Throat Spray | | sprays |

| **Medications taken every day** | | | | | |
| --- | --- | --- | --- | --- | --- |
| Medication Name  (Include strength if any) | How do you take this?  (Pill, injection, liquid, inhaler, transcutaneous/patch/topical, eye drop or ointment, ear drop, nasal spray, rectal) | How much do you take each time? (Include amount with unit) | How often do you take this?  (per day, week month, year) | How long have you been taking this?  (days, weeks, months, years) | Reason for Taking? |
|  |  |  |  |  |  |
|  |  |  |  |  |  |
|  |  |  |  |  |  |
|  |  |  |  |  |  |
|  |  |  |  |  |  |
|  |  |  |  |  |  |
|  |  |  |  |  |  |
|  |  |  |  |  |  |
|  |  |  |  |  |  |
|  |  |  |  |  |  |
|  |  |  |  |  |  |
|  |  |  |  |  |  |
|  |  |  |  |  |  |
|  |  |  |  |  |  |
|  |  |  |  |  |  |
|  |  |  |  |  |  |

| **Medications Taken As Needed** | | | | | |
| --- | --- | --- | --- | --- | --- |
| Medication Name  (Include strength if any) | How do you take this?  (Pill, injection, liquid, inhaler, transcutaneous/patch/topical, eye drop or ointment, ear drop, nasal spray, rectal) | How much do you take each time?  (Include amount with unit) | How often do you take this?  (per day, week month, year) | How long have you been taking this?  (days, weeks, months, years | Reason for Taking? |
|  |  |  |  |  |  |
|  |  |  |  |  |  |
|  |  |  |  |  |  |
|  |  |  |  |  |  |
|  |  |  |  |  |  |
|  |  |  |  |  |  |
|  |  |  |  |  |  |
|  |  |  |  |  |  |
|  |  |  |  |  |  |
|  |  |  |  |  |  |
|  |  |  |  |  |  |
|  |  |  |  |  |  |
|  |  |  |  |  |  |
|  |  |  |  |  |  |
|  |  |  |  |  |  |
|  |  |  |  |  |  |

**---------------------------------------------------------------------------------------------------------------------------------------------------**

**This is the end of the interview. Thank you for your cooperation. I am going to leave you some self-report surveys which I would now like you to complete. Do you have any questions?**

**---------------------------------------------------------------------------------------------------------------------------------------------------**

***(These last questions are for the interviewer, do not read to patient.)***

1. On a scale of 1-10, how distressed does this person currently seem to you?

0 1 2 3 4 5 6 7 8 9 10

Not At all Moderately Extremely

Distressed Distressed Distressed

2. How will the participant complete the self-report portion of the interview?

**0** ____ Laptop PC completed by participant

**1** ____ Laptop PC completed by interviewer (completed as interview, patient needs assistance)

**2** ____ Using paper surveys (internet is down or other) completed by participant

**3** ____ Using paper surveys (internet is down or other) completed by interviewer

2a. If 1 or 3 selected above, Why does participant need assistance with interview?

**0** ____ Immobilized on backboard

**1** ____ Other physical injury prevents use of computer

**2** ____ Needs help in understanding how to use computer or technology

3. How would you (the interviewer) rate the quality of the information obtained in this interview?

**0** ____ Excellent (Excellent effort and comprehension)

**1** ____ Good (Good effort and comprehension)

**2** ____Fair (Fair effort and comprehension)

**3** ____Poor (Poor effort and comprehension, overall quality of data is questionable)

**4** ____Inadequate (Interview terminated by interviewer, or quality felt to be too poor to be included in data set)

3a. If the quality of the information obtained in this interview was NOT excellent, what were the reasons? (Check all that apply)

**0** ____Respondent did not take interview seriously

**1**____Respondent did not understand the meaning of the questions

**2**____Interruptions or distractions

**3**____Respondent was offended by the interview

**4**____Respondent may have not been truthful because someone else was listening

**5**____Intoxication

**6**____Respondent was rushed

**7**____Interviewer not in respondent's native language

**8**____hearing (hearing loss or background noise)

**9**____Infirm (Age, weak, sick)

**10**____Other reason (specify) _____________________________________________________________

_____________________________________________________________________________________________________________________________________________________________________________________________________________________________________________

**RA Name/Initials: _________________________________________**

**Comments:**
