# Supplementary material for: Using emergency department-based inception cohorts to determine genetic characteristics associated with long term patient outcomes after motor vehicle collision: Methodology of the CRASH study
Source: BMC Emerg Med. 2011 Sep 26;11:14. doi: 10.1186/1471-227X-11-14 (PMC3188467; doi:10.1186/1471-227X-11-14)
Supplement: Additional file 2 — CRASH Methods ED Assessment Part 2. Part 2 of the 2 part survey administered in the Emergency Department. [file 1471-227X-11-14-S2.DOC]

**Thank you for taking the time to participate in Project CRASH. The information you provide will be very helpful to better understand how people recover after motor vehicle accidents. Please complete each of the following surveys. At the beginning of each survey, there are directions that explain how to answer the next group of questions. Please remember that your name and other identifying information will never be connected to the information you provide. If you have any questions please feel free to contact us. Thank you again for your participation!**

**__________________________________________________________________________________________**

***Your privacy is protected by a Certificate of Confidentiality from the National Institutes of Health (NIH). The researchers cannot be forced to disclose information that may identify you, even by a court subpoena, in any federal, state, or local civil, criminal, administrative, legislative, or other proceedings.***

**__________________________________________________________________________________________**

**The following questions have to do with your experience during and after the motor vehicle accident. Please choose the response that best describes how often you have felt or behaved this way during or after your motor vehicle accident.**

**0 = not at all true 1 = slightly true 2 = somewhat true 3 = very true 4 = extremely true**

**not somewhat extremely**

**Item Description** **true true true**

1. I felt helpless. **0** **1 2 3 4**

2. I felt sadness and grief. **0** **1 2 3 4**

3. I felt frustrated or angry. **0** **1 2 3 4**

4. I felt afraid for my safety. **0** **1 2 3 4**

5. I felt guilt. **0** **1 2 3 4**

6. I felt ashamed of my emotional reactions. **0** **1 2 3 4**

7. I felt worried about the safety of others. **0** **1 2 3 4**

8. I had the feeling I was about to lose

control of my emotions. **0** **1 2 3 4**

9. I had difficulty controlling my bowel

and bladder. **0** **1 2 3 4**

10. I was horrified by what happened. **0** **1 2 3 4**

11. I had physical reactions like sweating,

shaking, and pounding heart. **0** **1 2 3 4**

12. I felt I might pass out. **0** **1 2 3 4**

13. I thought I might die. **0** **1 2 3 4**

**Please choose the response that best describes how you felt during or after your motor vehicle accident.**

**strongly strongly**

**disagree neutral agree**

1. Compared to my normal experience, I

did not experience the normal **1** **2 3 4 5**

passage of time.

2. I felt as if I were in a daze. **1** **2 3 4 5**

3. It was as if I were watching myself. **1** **2 3 4 5**

4. I felt as if the events around the accident

were happening to someone else. **1** **2 3 4 5**

5. I felt as if I were in a dream. **1** **2 3 4 5**

**These questions are about your beliefs concerning who was at fault for the accident. Please select the response that best corresponds to your answer.**

1. Which statement best describes your feeling about the accident? (select one)

**1** ____ It was my fault.

**2** ____ It was another person’s fault.

**3** ____ It was nobody’s fault. (Bad weather, or hard to avoid for another reason.)

2. **If it was another person’s fault**, how angry or upset are you at them right now? (select one)

**1** ____ Not at all

**2** ____ A little

**3** ____ Somewhat

**4** ____ A lot

**These questions ask for your views about your health before the motor vehicle accident. Please answer every question. If you are unsure about how to answer a question, please give the best answer you can.**

**1. In general, thinking about your health before the accident, would you say your health was:**

**1____** Excellent

**2____** Very good

**3____** Good

**4____** Fair

**5____** Poor

**2. The following questions are about activities you might do during a typical day. Did your health before the accident limit you in these activities? If so, how much?**

**1 = Yes, Limited a Lot 2 = Yes, Limited a Little 3 = No, Not Limited at All**

**Lot Little Not at all**

**Moderate activities**, such as moving a table, pushing

a vacuum cleaner, bowling, or playing golf **1 2 3**

Climbing **several** flights of stairs **1 2 3**

**3. During the past four weeks, how much of the time have you had any of the following problems with your work or other regular daily activities *as a result of your physical health*?**

**1 = all of the time 2 = most of the time 3 = some of the time 4 = a little of the time 5 = none of the time**

**all of most some little none of**

**the time the time**

**Accomplished less** than you would like **1 2 3 4 5**

Were limited in the **kind** of work or

other activities **1 2 3 4 5**

**4. During the past four weeks, how much of the time have you had any of the following problems with your work or other regular daily activities *as a result of any emotional problems* (such as feeling depressed or anxious)?**

**1 = all of the time 2 = most of the time 3 = some of the time 4 = a little of the time 5 = none of the time**

**all of most some little none of**

**the time the time**

**Accomplished less** than you

would like **1 2 3 4 5**

Did work or other activities **less**

**carefully than usual** **1 2 3 4 5**

**5.** **During the past 4 weeks, how much did pain interfere with your normal work (including both work outside the home and housework)?**

**1____** Not at all

**2____** A little bit

**3____** Moderately

**4____** Quite a bit

**5____** Extremely

**6. These questions are about how you feel and how things have been with you during the past 4 weeks. For each question, please give the one answer that comes closest to the way you have been feeling.**

**1 = all of the time 2 = most of the time 3 = some of the time 4 = a little of the time 5 = none of the time**

**all of most some little none of**

**the time the time**

Have you felt calm and peaceful **1 2 3 4 5**

Did you have a lot of energy **1 2 3 4 5**

Have you felt downhearted

and depressed **1 2 3 4 5**

**7. During the past 4 weeks, how much of the time has your physical health or emotional problems interfered with your social activities (like visiting friends, relatives, etc.)?**

**1____** All of the time

**2____** Most of the time

**3____** Some of the time

**4____** A little of the time

**5____** None of the time

**These questions are about how you have felt or behaved during the PAST WEEK. Please, select the response that best corresponds to your answer.**

| **In the Past Week**: | **Rarely or none of the time  (less than 1 day)** | **Some or a little of the time (1-2 days)** | **Occasionally or a moderate amount of the time (3-4 days)** | **Most or all of the time (5-7 days)** |
| --- | --- | --- | --- | --- |
| 1. I was bothered by things that usually don’t bother me. |  |  |  |  |
| 2. I did not feel like eating: my appetite was poor. |  |  |  |  |
| 3. I felt that I could not shake off the blues even with help from my family or friends. |  |  |  |  |
| 4. I felt that I was just as good as other people. |  |  |  |  |
| 5. I had trouble keeping my mind on what I was doing. |  |  |  |  |
| 6. I felt depressed. |  |  |  |  |
| 7. I felt that everything I did was an effort. |  |  |  |  |
| 8. I felt hopeful about the future. |  |  |  |  |
| 9. I thought my life had been a failure. |  |  |  |  |
| 10. I felt fearful. |  |  |  |  |
| 11. My sleep was restless. |  |  |  |  |
| 12. I was happy. |  |  |  |  |
| 13. I talked less than usual. |  |  |  |  |
| 14. I felt lonely. |  |  |  |  |
| 15. People were unfriendly. |  |  |  |  |
| 16. I enjoyed life. |  |  |  |  |
| 17. I had crying spells. |  |  |  |  |
| 18. I felt sad. |  |  |  |  |
| 19. I felt that people disliked me. |  |  |  |  |
| 20. I could not get going. |  |  |  |  |

**A number of statements that people use to describe themselves are given below. Read each statement, and then indicate how you GENERALLY FEEL. There are no right or wrong answers. Do not spend too much time on any one statement but choose the answer which seems to describe how you GENERALLY FEEL.**

| **HOW I GENERALLY FEEL:** | **Almost Never** | **Sometimes** | **Often** | **Almost Always** |
| --- | --- | --- | --- | --- |
| 1. I am a steady person. |  |  |  |  |
| 2. I am quick-tempered. |  |  |  |  |
| 3. I feel satisfied with myself. |  |  |  |  |
| 4. I have a fiery temper. |  |  |  |  |
| 5. I get in a state of tension or turmoil as I think over my recent concerns and interests. |  |  |  |  |
| 6. I am a hot-headed person. |  |  |  |  |
| 7. I wish I could be as happy as others seem to be. |  |  |  |  |
| 8. I get angry when I’m slowed down by others’ mistakes. |  |  |  |  |
| 9. I feel like a failure. |  |  |  |  |
| 10. I feel annoyed when I am not given recognition for doing good work. |  |  |  |  |
| 11. I feel nervous and restless. |  |  |  |  |
| 12. I fly off the handle. |  |  |  |  |
| 13. I feel secure. |  |  |  |  |
| 14. When I get mad, I say nasty things. |  |  |  |  |
| 15. I lack self-confidence. |  |  |  |  |
| 16. It makes me furious when I am criticized in front of others. |  |  |  |  |
| 17. I feel inadequate. |  |  |  |  |
| 18. When I get frustrated, I feel like hitting someone. |  |  |  |  |
| 19. I worry too much over something that really does not matter. |  |  |  |  |
| 20. I feel infuriated when I do a good job and get a poor evaluation. |  |  |  |  |

**These questions collect information about you and your life. Please select the response or responses that best correspond to your answer.**

1. Do you currently either go to school or work outside the home? Select all that apply.

**1** ____ I do not work outside the home

**2** ____ Student

**3** ____ Work part time

**4** ____ Work full time

**5** ____ Disabled from work and receiving disability

**6** ____ Disabled from work and disability pending

**7** ____ Disabled from work and do not have disability

**8** ____ Don’t know or refused to answer

2. What is your Marital or relationship status at this time? Select all that apply.

**1** ____ No serious relationship at this time

**2** ____ Serious relationship, not living together

**3** ____ Serious relationship, living together

**4** ____ Married

**5**____ Don’t know or refused to answer

3. What is the highest grade or level of schooling that you have completed? Select one option.

**1** ____ Less than 8 years

**2** ____ 8-11 years

**3** ____ 12 years or completed high school

**4** ____ Post high school training other than college

**5** ____ Some college

**6** ____ College graduate

**7** ____ Post graduate level

**8** ____ Refuse

**9** ____ Don’t know

4. What is your family’s current annual household income? Please include all sources of income for all family members such as wages, salaries, investments, etc. Select one option.

**1** ____ $0-$19,999

**2** ____ $20,000-$39,999

**3** ____ $40,000-$59,999

**4** ____ $60,000-$79,999

**5** ____ $80,000-$99,000

**6** ____ $100,000-149,999

**7** ____ $150,000 or higher

**8** ____ Refuse

**9** ____ Don’t know

**Please complete the following questions about your ethnicity by selecting the best response.**

Maternal Grandmother- Mom’s Mom Maternal Grandfather- Mom’s Dad

Paternal Grandmother- Dad’s Mom Paternal Grandfather- Dad’s Dad

5. My mother’s ethnicity is: (select one option)

**1 ____** Hispanic or Latino **3 ____** Refuse

**2 ____** Not Hispanic or Latino **4 ____** Don’t Know

6. My father’s ethnicity is: (select one option)

**1 ____** Hispanic or Latino **3 ____** Refuse

**2 ____** Not Hispanic or Latino **4 ____** Don’t Know

7. My maternal grandmother’s ethnicity is: (select one option)

**1 ____** Hispanic or Latino **3 ____** Refuse

**2 ____** Not Hispanic or Latino **4 ____** Don’t Know

8. My paternal grandmother’s ethnicity is: (select one option)

**1 ____** Hispanic or Latino **3 ____** Refuse

**2 ____** Not Hispanic or Latino **4 ____** Don’t Know

9. My maternal grandfather’s ethnicity is: (select one option)

**1 ____** Hispanic or Latino **3 ____** Refuse

**2 ____** Not Hispanic or Latino **4 ____** Don’t Know

10. My paternal grandfather’s ethnicity is: (select one option)

**1 ____** Hispanic or Latino **3 ____** Refuse

**2 ____** Not Hispanic or Latino **4 ____** Don’t Know

11. My mother’s race is: (select all that apply)

**1 ____** American Indian or Alaska Native **7 ____** Don’t Know

**2 ____** Asian **8 ____** Refuse

**3 ____** Black or African American

**4 ____** Native Hawaiian or Other Pacific

**5 ____** White

**6 ____** Other (please specify): _______________________________

12. My father’s race is: (select all that apply)

**1 ____** American Indian or Alaska Native **7 ____** Don’t Know

**2 ____** Asian **8 ____** Refuse

**3 ____** Black or African American

**4 ____** Native Hawaiian or Other Pacific

**5 ____** White

**6 ____** Other (please specify): _______________________________

13. My maternal grandmother’s race is: (select all that apply)

**1 ____** American Indian or Alaska Native **7 ____** Don’t Know

**2 ____** Asian **8 ____** Refuse

**3 ____** Black or African American

**4 ____** Native Hawaiian or Other Pacific

**5 ____** White

**6 ____** Other (please specify): _______________________________

14. My paternal grandmother’s race is: (select all that apply)

**1 ____** American Indian or Alaska Native **7 ____** Don’t Know

**2 ____** Asian **8 ____** Refuse

**3 ____** Black or African American

**4 ____** Native Hawaiian or Other Pacific

**5 ____** White

**6 ____** Other (please specify): _______________________________

15. My maternal grandfather’s race is: (select all that apply)

**1 ____** American Indian or Alaska Native **7 ____** Don’t Know

**2 ____** Asian **8 ____** Refuse

**3 ____** Black or African American

**4 ____** Native Hawaiian or Other Pacific

**5 ____** White

**6 ____** Other (please specify): _______________________________

16. My paternal grandfather’s race is: (select all that apply)

**1 ____** American Indian or Alaska Native **7 ____** Don’t Know

**2 ____** Asian **8 ____** Refuse

**3 ____** Black or African American

**4 ____** Native Hawaiian or Other Pacific

**5 ____** White

**6 ____** Other (please specify): _______________________________

**Please enter the following information.**

17. What is your height?

 feet   inches

18. What is your weight?

   pounds

**Below are 10 statements that you may agree or disagree with. Using the scale below, indicate your agreement with each item. Be open and honest in your responding.**

0 = Strongly Disagree

1 = Disagree

2 = Neutral

3 = Agree

4 = Strongly Agree

_____ **1)** In uncertain times, I usually expect the best.

_____ **2)** It’s easy for me to relax.

**_____ 3)** If something can go wrong for me, it will.

**_____ 4)** I'm always optimistic about my future.

**_____ 5)** I enjoy my friends a lot.

**_____ 6)** It's important for me to keep busy.

**_____ 7)** I hardly ever expect things to go my way.

**_____ 8)** I don't get upset too easily.

**_____ 9)** I rarely count on good things happening to me.

**_____ 10)** Overall, I expect more good things to happen to me than bad.

**Everyone experiences painful situations at some point in their lives. Such experiences may include headaches, tooth pain, joint or muscle pain. People are often exposed to situations that may cause pain, such as illness, injury, dental procedures, or surgery. We are interested in the types of thoughts and feelings that you have when you are in pain. Listed below are 13 statements describing different thoughts and feelings that may be associated with pain. Using the following scale, please indicate the degree to which you have these thoughts and feelings when you are experiencing pain.**

| **0 = not at all** | **1 = to a slight degree** | **2 = to a moderate degree** | **3 = to a great degree** | **4 = all the time** |
| --- | --- | --- | --- | --- |

**Not at slight moderate great all**

**all degree degree degree the time**

1. I worry all the time about whether it will end. **0 1 2 3 4**

2. I feel I can't go on. **0 1 2 3 4**

3. It's terrible and I think it's never going to get any better. **0 1 2 3 4**

4. It's awful and I feel that it overwhelms me. **0 1 2 3 4**

5. I feel I can't stand it anymore. **0 1 2 3 4**

6. I become afraid that the pain will get worse. **0 1 2 3 4**

7. I keep thinking of other painful events. **0 1 2 3 4**

8. I anxiously want the pain to go away. **0 1 2 3 4**

9. I can't seem to get it out of my mind. **0 1 2 3 4**

10. I keep thinking about how much it hurts. **0 1 2 3 4**

11. I keep thinking about how badly I want the pain to stop. **0 1 2 3 4**

12. There's nothing I can do to reduce the intensity of the pain. **0 1 2 3 4**

13. I wonder whether something serious may happen. **0 1 2 3 4**

**Read each of the following statements carefully. Indicate how you feel about each statement using the following scale.**

**1= Very strongly disagree**

**2= Strongly disagree**

**3= Mildly disagree**

**4= You are neutral**

**5= Mildly agree**

**6= Strongly agree**

**7= Very strongly agree**

1. There is a special person who is around when you are in need. _____ (1-7)

2. There is a special person with whom you can share your joys and sorrows. _____ (1-7)

3. You have a special person who is a real source of comfort to you. _____ (1-7)

4 .There is a special person in your life who cares about your feelings. _____ (1-7)

**These questions ask about your use of alcohol, tobacco, and other substances. Please select the response that best indicates your answer or, if indicated, directly enter your answer.**

1. Do you smoke cigarettes?

0 ____ No

1 ____ Yes

1a. If so, how may cigarettes do you smoke per day on average?

   (1 pack = 20 cigarettes)

2. Have you ever drank alcohol?

0 ____ No *(skip to question 10)*

1 ____ Yes

3. **During the past 4 weeks**, on average, how many days per week have you had any alcohol to drink (for example, beer, wine, or any drink containing liquor)?

____ **0** or none **(*if none, skip ahead to question 5*)**

____ **1**

____ **2**

____ **3**

____ **4**

____ **5**

____ **6**

____ **7** or every day

2b. **During the past 4 weeks**, on the days that you drank, how much did you usually drink? *(by drink, we mean one 12 oz. can or bottle of beer, or one 5 oz. glass of wine, or a drink containing a 1 oz. shot of liquor)?*

  /day

4. **During the past 4 weeks**, how many heavy drinking days have you had? Heavy drinking means 6 drinks or more in one day for men and 4 drinks or more in one day for women. *(by drink, we mean one 12 oz. can or bottle of beer, or one 5 oz. glass of wine, or a drink containing a 1 oz. shot of liquor)?*

 days (0-28)

5. How many drinks does it take before you begin to feel the first effects of the alcohol? *(by drink, we mean one 12 oz. can or bottle of beer, or one 5 oz. glass of wine, or one a drink containing a 1 oz. shot of liquor)*

  drinks

6. Have close friends or relatives worried or complained about your drinking in the past year?

**0** ____ No

**1** ____ Yes

7. Do you sometimes take a drink in the morning when you first get up?

**0** ____ No

**1** ____ Yes

8. Are there times when you drink and afterwards you can’t remember what you said or did?

**0** ____ No

**1** ____ Yes

9. Do you sometimes feel the need to cut down on your drinking?

**0** ____ No

**1** ____ Yes

10. **During the past 3 months**, have you ever used **marijuana or hash**?

**0** ____ No

**1** ____ Yes

10a. **If yes**, about how many days have you used marijuana or hash **during the past 3 months**?

 days (0-90)

11. **During the past 3 months**, have you ever used **cocaine or crack**?

**0** ____ No

**1** ____ Yes

11a. **If yes**, about how many days have you used cocaine or crack **during the past 3 months**?

 days (0-90)

12. **During the past 3 months**, have you ever used **heroin**?

**0** ____ No

**1** ____ Yes

12a. **If yes**, about how many days have you used heroin **during the past 3 months**?

  days (0-90)

1. Is there anything else that you would like to tell us? Is there anything else that we haven’t asked you, which is important for us to understand about the experience of being in a motor vehicle collision?

__________________________________________________________________________________________________________________________________________________________________________________________________________________________________________________________________________________________________________________________________________________________________________________________________________________________________________________________________

---------------------------------------------------------------------------------------------------------------------------

**Thank you for completing these surveys!**

**---------------------------------------------------------------------------------------------------------------------------**
