# Supplementary material for: Using emergency department-based inception cohorts to determine genetic characteristics associated with long term patient outcomes after motor vehicle collision: Methodology of the CRASH study
Source: BMC Emerg Med. 2011 Sep 26;11:14. doi: 10.1186/1471-227X-11-14 (PMC3188467; doi:10.1186/1471-227X-11-14)
Supplement: Additional file 3 — CRASH Methods Follow-Up Questionnaire. The survey administered at each follow-up interval. [file 1471-227X-11-14-S3.DOC]

**Thank you for taking the time to participate in Project CRASH. The information you provide will be very helpful to better understand how people recover after motor vehicle accidents. Please complete all of the following questions. At the beginning of each group of questions, there are directions about how to answer them. Thank you again for your participation. If you have any questions please feel free to contact us.**

**__________________________________________________________________________________________**

***Your privacy is protected by a Certificate of Confidentiality from the National Institutes of Health (NIH). The researchers cannot be forced to disclose information that may identify you, even by a court subpoena, in any federal, state, or local civil, criminal, administrative, legislative, or other proceedings.***

**__________________________________________________________________________________________**

**Missed Work or Usual Activities**

**These questions are about any difficulty with work or usual activities after your accident.**

1. After your emergency department visit on (*date)*, did **you miss any work** because of injuries or other health problems that were **caused by your motor vehicle accident?**

**0** ____ No

**1** ____ Yes, missed work

**2** ____ Not currently working a paid job

1a. **If yes**, how much time did you miss?

 days/ weeks/months/years

2. After your emergency department visit on (*date)*, **were you unable to perform your usual activities** because of injuries or other health problems that were **caused by your motor vehicle accident?**

**0** ____ No

**1** ____ Yes

2a. **If yes**, how much time were you unable to perform your usual activities?

 days/ weeks/months/years

**Regional Pain Scale**

**The following questions are about the amount of pain and/or tenderness that you have had over THE PAST 7 DAYS in each of the body areas listed below. Please select the response that corresponds to the answer that best describes your pain or tenderness on a scale from 0-10, where 0 is no pain or tenderness and 10 is pain or tenderness as severe as it could be. Also, for each body area where you are having pain, please indicate whether this pain is related to your motor vehicle accident.**

Is this pain related to your motor vehicle accident?

**NO SEVERE**

**PAIN PAIN**

3. Head 0 1     2     3     4     5     6     7     8     9    10 ____NO ____YES

4. Neck 0 1     2     3     4     5     6     7     8     9    10 ____NO ____YES

5. Left Jaw 0 1     2     3     4     5     6     7     8     9    10 ____NO ____YES

6. Right Jaw 0 1     2     3     4     5     6     7     8     9    10 ____NO ____YES

7. Left Shoulder 0 1     2     3     4     5     6     7     8     9    10 ____NO ____YES

8. Right Shoulder 0 1     2     3     4     5     6     7     8     9    10 ____NO ____YES

9. Left Upper Arm 0 1     2     3     4     5     6     7     8     9    10 ____NO ____YES

10. Right Upper Arm 0 1     2     3     4     5     6     7     8     9    10 ____NO ____YES

11. Left Lower Arm 0 1     2     3     4     5     6     7     8     9    10 ____NO ____YES

12. Right Lower Arm 0 1     2     3     4     5     6     7     8     9    10 ____NO ____YES

13. Chest 0 1     2     3     4     5     6     7     8     9    10 ____NO ____YES

14. Upper Back 0 1     2     3     4     5     6     7     8     9    10 ____NO ____YES

15. Lower Back 0 1     2     3     4     5     6     7     8     9    10 ____NO ____YES

16. Abdomen 0 1     2     3     4     5     6     7     8     9    10 ____NO ____YES

17. Left Hip 0 1     2     3     4     5     6     7     8     9    10 ____NO ____YES

18. Right Hip 0 1     2     3     4     5     6     7     8     9    10 ____NO ____YES

19. Left Upper Leg 0 1     2     3     4     5     6     7     8     9    10 ____NO ____YES

20. Right Upper Leg 0 1     2     3     4     5     6     7     8     9    10 ____NO ____YES

21. Left Lower Leg 0 1     2     3     4     5     6     7     8     9    10 ____NO ____YES

22. Right Lower Leg 0 1     2     3     4     5     6     7     8     9    10 ____NO ____YES

23. On a scale of zero to ten, where zero means no pain and ten equals pain as severe as it could possibly be, what is the usual intensity of your pain during **THE PAST 7 DAYS,** considering any or all of your pains together?

***Record 0-10 response ________ (allow and record “.5” responses)***

**Somatic Symptoms (SILL)**

The following questions ask about any problems you may have had with other symptoms in **THE PAST MONTH**. Please select the response that best describes how much of a problem you have had in **THE PAST MONTH** with the following symptoms, where zero means no problem and 10 means a major problem.

**NO MAJOR**

**PROBLEM PROBLEM**

24. Headaches 0 1         2          3          4          5          6         7         8          9         10

25. Dizziness 0 1         2          3          4          5          6         7         8          9         10

26. Nausea 0 1         2          3          4          5          6         7         8          9         10

27. Noise Sensitivity 0 1         2          3          4          5          6         7         8          9         10

28. Light Sensitivity 0 1         2          3          4          5          6         7         8 9 10

29. Concentration Difficulty 0 1         2          3          4          5          6         7         8          9         10

30. Taking longer to think 0 1         2          3          4          5          6         7         8          9         10

31. Blurred Vision 0 1         2          3          4          5          6         7         8          9         10

32. Double Vision 0 1         2          3          4          5          6         7         8          9         10

33. Restlessness 0 1         2          3          4          5          6         7         8          9         10

34. Upset Stomach 0 1         2          3          4          5          6         7         8          9         10

35. Persistent Fatigue 0 1         2          3          4          5          6         7         8          9         10

36. Sensitive or tender skin 0 1         2          3          4          5          6         7         8          9         10

37. Ringing in ears 0 1         2          3          4          5          6         7         8          9         10

38. Itchy eyes or skin 0 1         2          3          4          5          6         7         8          9         10

39. Racing heart 0 1         2          3          4          5          6         7         8          9         10

40. Insomnia or difficulty 0 1         2          3          4          5          6         7         8          9         10

sleeping

41. Hands trembling or shaking 0 1         2          3          4          5          6         7         8          9         10

42. Feeling faint 0 1         2          3          4          5          6         7         8          9         10

43. Abdominal pain 0 1         2          3          4          5          6         7         8          9         10

44. Constipation and/or 0 1         2          3          4          5          6         7         8          9         10

diarrhea

**Pain Interference Subscale from Brief Pain Inventory**

**The following questions are about how pain resulting from your accident has interfered with your life during the past week. Please select the response that corresponds to the number that best describes how pain resulting from your motor vehicle accident has interfered, where 0 is no interference and 10 is complete interference.**

**During the past week, how much has pain resulting from your accident interfered with your:**

45. **General Activity** 0 1 2 3 4 5 6 7 8 9 10

Does not Completely

Interfere Interferes

46. **Mood** 0 1 2 3 4 5 6 7 8 9 10

Does not Completely

Interfere Interferes

47. **Walking Ability** 0 1 2 3 4 5 6 7 8 9 10

Does not Completely

Interfere Interferes

48. **Normal Work** *(includes both work outside the home and housework)*

0 1 2 3 4 5 6 7 8 9 10

Does not Completely

Interfere Interferes

49. **Relations with other people**

0 1 2 3 4 5 6 7 8 9 10

Does not Completely

Interfere Interferes

50. **Sleep** 0 1 2 3 4 5 6 7 8 9 10

Does not Completely

Interfere Interferes

51. **Enjoyment of life** 0 1 2 3 4 5 6 7 8 9 10

Does not Completely

Interfere Interferes

**New or Re-Injury Questions**

**The next questions ask about any new injury or illness you might have had since your motor vehicle accident.**

52. After your emergency department visit on (*date)*, have you had a **NEW neck injury**, NOT RELATED to your motor vehicle accident, that required you to go to the Emergency Room, Hospital, or Doctor’s Office?

**0** ____ No

**1** ____ Yes

52a. **If yes**, how did it happen?

**______________________________________________________________________________________________________________________________________________________________________________________________________________________________________________________________________________________________________**

52b. **If yes**, what treatment did you have for it? (select all that apply)

**0** ____ Medications

**1** ____ Surgery

**2**____ Physical Therapy

**3**____ Other (list: ___________________________________________________________________)

53. After your emergency department visit on (*date)*, have you had a **NEW back injury**, NOT RELATED to your motor vehicle accident, that required you to go to the Emergency Room, Hospital, or Doctor’s Office?

**0** ____ No

**1** ____ Yes

53a. **If yes**, how did it happen?

**______________________________________________________________________________________________________________________________________________________________________________________________________________________________________________________________________________________________________**

53b. **If yes**, what treatment did you have for it? (select all that apply)

**0** ____Medications

**1** ____Surgery

**2**____ Physical Therapy

**3**____ Other (list: ___________________________________________________________________)

54. After your emergency department visit on (*date)*, have you had any new **significant health problem** develop, **NOT RELATED** to your motor vehicle accident, that required you to go to the Emergency Room, Hospital, or Doctor’s Office?

**0** ____ No

**1** ____ Yes

54a. **If yes**, please describe?

**______________________________________________________________________________________________________________________________________________________________________________________________________________________________________________________________________________________________________**

**Center for Epidemiological Studies Depression Scale**

**These questions are about how you have felt or behaved during the PAST WEEK. Please select the response that best corresponds to your answer.**

| **In the Past Week**: | **Rarely or none of the time  (less than 1 day)** | **Some or a little of the time (1-2 days)** | **Occasionally or a moderate amount of the time (3-4 days)** | **Most or all of the time (5-7 days)** |
| --- | --- | --- | --- | --- |
| 55. I was bothered by things that usually don’t bother me. |  |  |  |  |
| 56. I did not feel like eating: my appetite was poor. |  |  |  |  |
| 57. I felt that I could not shake off the blues even with help from my family or friends. |  |  |  |  |
| 58. I felt that I was just as good as other people. |  |  |  |  |
| 59. I had trouble keeping my mind on what I was doing. |  |  |  |  |
| 60. I felt depressed. |  |  |  |  |
| 61. I felt that everything I did was an effort. |  |  |  |  |
| 62. I felt hopeful about the future. |  |  |  |  |
| 63. I thought my life had been a failure. |  |  |  |  |
| 64. I felt fearful. |  |  |  |  |
| 65. My sleep was restless. |  |  |  |  |
| 66. I was happy. |  |  |  |  |
| 67. I talked less than usual. |  |  |  |  |
| 68. I felt lonely. |  |  |  |  |
| 69. People were unfriendly. |  |  |  |  |
| 70. I enjoyed life. |  |  |  |  |
| 71. I had crying spells. |  |  |  |  |
| 72. I felt sad. |  |  |  |  |
| 73. I felt that people disliked me. |  |  |  |  |
| 74. I could not get going. |  |  |  |  |

**State-Trait Personality Inventory (Form Y)**

**The following are a number of statements that people use to describe themselves. For each statement, please indicate how each statement relates to how you GENERALLY FEEL. There are no right or wrong answers. Do not spend too much time on any one statement but choose the answer which seems to describe how you GENERALLY FEEL.**

| **HOW I GENERALLY FEEL:** | **Almost Never** | **Sometimes** | **Often** | **Almost Always** |
| --- | --- | --- | --- | --- |
| 75. I am a steady person. |  |  |  |  |
| 76. I am quick-tempered. |  |  |  |  |
| 77. I feel satisfied with myself. |  |  |  |  |
| 78. I have a fiery temper. |  |  |  |  |
| 79. I get in a state of tension or turmoil as I think over my recent concerns and interests. |  |  |  |  |
| 80. I am a hot-headed person. |  |  |  |  |
| 81. I wish I could be as happy as others seem to be. |  |  |  |  |
| 82. I get angry when I’m slowed down by others’ mistakes. |  |  |  |  |
| 83. I feel like a failure. |  |  |  |  |
| 84. I feel annoyed when I am not given recognition for doing good work. |  |  |  |  |
| 85. I feel nervous and restless. |  |  |  |  |
| 86. I fly off the handle. |  |  |  |  |
| 87. I feel secure. |  |  |  |  |
| 88. When I get mad, I say nasty things. |  |  |  |  |
| 89. I lack self-confidence. |  |  |  |  |
| 90. It makes me furious when I am criticized in front of others. |  |  |  |  |
| 91. I feel inadequate. |  |  |  |  |
| 92. When I get frustrated, I feel like hitting someone. |  |  |  |  |
| 93. I worry too much over something that really does not matter. |  |  |  |  |
| 94. I feel infuriated when I do a good job and get a poor evaluation. |  |  |  |  |

**Travel Anxiety Questions (From Mayou)**

**The next two questions ask about any concerns you may have about being in a motor vehicle after your accident. Please select the response that corresponds to the answer that best describes your feelings.**

95.) How do you feel about driving in a motor vehicle now compared to before the accident? Please select one option.

**1** ______ About the same as before the accident

**2** ______ A little more nervous than before the accident

**3** ______ Quite a bit more nervous than before the accident

**4** ______ Much more nervous than before the accident

**5** ______ Not applicable (don’t drive)

96.) How do you feel about being a passenger in a motor vehicle with another driver now compared to before the accident? Please select one option.

**1** ______ About the same as before the accident

**2** ______ A little more nervous than before the accident

**3** ______ Quite a bit more nervous than before the accident

**4** ______ Much more nervous than before the accident

# Impact of Events Scale – Revised

# The following is a list of difficulties people sometimes have after motor vehicle accidents. For each item, please indicate how distressing each difficulty has been for you, during the past 7 days, because of your motor vehicle accident. How much were you bothered by these difficulties during the past 7 days?

| The scale is: | Not  at  all | A  little  bit | Moderately | Quite  a bit | Extremely |
| --- | --- | --- | --- | --- | --- |
| 97. Any reminder of the accident brought back feelings about it. | 0 | 1 | 2 | 3 | 4 |
| 98. You had trouble staying asleep. | 0 | 1 | 2 | 3 | 4 |
| 99. Other things kept making you think about the accident. | 0 | 1 | 2 | 3 | 4 |
| 100. You felt irritable and angry. | 0 | 1 | 2 | 3 | 4 |
| 101. You avoided letting yourself get upset when you thought about the accident or were reminded of it. | 0 | 1 | 2 | 3 | 4 |
| 102. You thought about the accident when you didn’t mean to. | 0 | 1 | 2 | 3 | 4 |
| 103. You felt as if the accident hadn’t happened or wasn’t real. | 0 | 1 | 2 | 3 | 4 |
| 104. You stayed away from reminders about the accident. | 0 | 1 | 2 | 3 | 4 |
| 105. Pictures about the accident popped into your mind. | 0 | 1 | 2 | 3 | 4 |
| 106. You were jumpy and easily startled. | 0 | 1 | 2 | 3 | 4 |
| 107. You tried not to think about the accident. | 0 | 1 | 2 | 3 | 4 |
| 108. You were aware that you still had a lot of feelings about the accident, but you didn’t deal with them. | 0 | 1 | 2 | 3 | 4 |
| 109. Your feelings about the accident were kind of numb. | 0 | 1 | 2 | 3 | 4 |
| 110. You found yourself acting or feeling like you were back at that time. | 0 | 1 | 2 | 3 | 4 |
| 111. You had trouble falling asleep. | 0 | 1 | 2 | 3 | 4 |
| 112. You had waves of strong feelings about the accident. | 0 | 1 | 2 | 3 | 4 |
| 113. You tried to remove the accident from your memory. | 0 | 1 | 2 | 3 | 4 |
| 114. You had trouble concentrating. | 0 | 1 | 2 | 3 | 4 |
| 115. Reminders of the accident caused you to have physical reactions, such as sweating, trouble breathing, nausea or a pounding heart. | 0 | 1 | 2 | 3 | 4 |
| 116. You had dreams about the accident | 0 | 1 | 2 | 3 | 4 |
| 117. You felt watchful and on guard. | 0 | 1 | 2 | 3 | 4 |
| 118. You tried not to talk about it. | 0 | 1 | 2 | 3 | 4 |

**SF-12 v2**

**These questions ask for your views about your health. Please, answer every question by selecting the response that best corresponds with your answer. If you are unsure about how to answer a question, please give the best answer you can.**

**119. In general, would you say your health is:**

**1____** Excellent

**2____** Very good

**3____** Good

**4____** Fair

**5____** Poor

**120. The following questions are about activities you might do during a typical day. Does your health now limit you in these activities? If so, how much?**

**1 = Yes, Limited a Lot 2 = Yes, Limited a Little 3 = No, Not Limited at All**

**Lot Little Not at all**

**Moderate activities**, such as moving a table, pushing

a vacuum cleaner, bowling, or playing golf **1 2 3**

Climbing **several** flights of stairs **1 2 3**

**121. During the past four weeks, how much of the time have you had any of the following problems with your work or other regular daily activities *as a result of your physical health*?**

**all of most some little none of**

**the time the time**

**Accomplished less** than you would like **1 2 3 4 5**

Were limited in the **kind** of work or

other activities **1 2 3 4 5**

**122. During the past four weeks, how much of the time have you had any of the following problems with your work or other regular daily activities *as a result of any emotional problems*  (such as feeling depressed or anxious)?**

**1 = all of the time 2 = most of the time 3 = some of the time 4 = a little of the time 5 = none of the time**

**all of most some little none of**

**the time the time**

**Accomplished less** than you

would like **1 2 3 4 5**

Did work or other activities **less**

**carefully than usual** **1 2 3 4 5**

**123.** **During the past 4 weeks, how much did pain interfere with your normal work (including both work outside the home and housework)?**

**1____** Not at all

**2____** A little bit

**3____** Moderately

**4____** Quite a bit

**5____** Extremely

**124. These questions are about how you feel and how things have been with you during the past 4 weeks. For each question, please give the one answer that comes closest to the way you have been feeling.**

**1 = all of the time 2 = most of the time 3 = some of the time 4 = a little of the time 5 = none of the time**

**all of most some little none of**

**the time the time**

Have you felt calm and peaceful **1 2 3 4 5**

Did you have a lot of energy **1 2 3 4 5**

Have you felt downhearted

and depressed **1 2 3 4 5**

**125. During the past 4 weeks, how much of the time has your physical health or emotional problems interfered with your social activities (like visiting friends, relatives, etc.)?**

**1____** All of the time

**2____** Most of the time

**3____** Some of the time

**4____** A little of the time

**5____** None of the time

**Health Service Utilization**

**The next questions are about any doctor’s visits or treatments that you may have had because of your motor vehicle accident.**

1. Since your emergency department visit, have you had any **additional or follow-up** **testing**, such as x-rays or blood work, because of injuries or other health problems that were **related to your motor vehicle accident**?

**0** ____ No

**1** ____ Yes

**If yes**, please select the testing you have had from the following list:

126a. **MRI 0** ____ No **1** ____ Yes

126b. If yes, please select the area of the body

**0** ____ Head

**1** ____ Neck

**2** ____ Chest

**3** ____ Abdomen

**4** ____ Pelvic/Hip

**5** ____ Arms

**6** ____ Legs

126c. **X-ray**  **0** ____ No **1** ____ Yes

128d. If yes, please select the area of the body

**0** ____ Head

**1** ____ Neck

**2** ____ Chest

**3** ____ Abdomen

**4** ____ Pelvic/Hip

**5** ____ Arms

**6** ____ Legs

126e. **CT scan 0** ____ No **1** ____ Yes

126f. If yes, please select the area of the body

**0** ____ Head

**1** ____ Neck

**2** ____ Chest

**3** ____ Abdomen

**4** ____ Pelvic/Hip

**5** ____ Arms

**6** ____ Legs

126g. **Blood work /Labs 0** ____ No **1** ____ Yes

126e. **Other** (please specify) **0** ____ No **1** ____ Yes

_____________________________________________________________________________________ _____________________________________________________________________________________

1. Since your emergency department visit, have you been to any **medical** **professionals** because of injuries or other health problems that were **related to your motor vehicle accident**?

**0** ____ No

**1** ____ Yes

1. **If yes**, what type of professional did you see? Please choose from the following list:

128a **Primary Care** Y/N **If yes, how many times?**

____Family Physician/General Doctor ______

____Internal Medicine ______

128b**. Surgeon** Y/N

____General Surgeon ______

____Orthopedic Surgeon ______

____Plastic Surgeon ______

____Trauma Surgeon ______

128c. **Mental Health Specialist** Y/N

____Psychiatrist ______

____Psychologist ______

____Social Worker ______

128d. **Other Specialist** Y/N

____Orthopedist ______

____Physical Therapist ______

____Physical Medicine Specialist/Physiatrist/PMNR ______

____Chiropractor ______

____Neurologist ______

____ENT ______

____Massage/Manual Therapist ______

128e. **Alternative Medicine** Y/N

____Acupuncturist ______

____Herbalist ______

____Naturopath ______

____Homeopath ______

____Reiki/Energy Medicine Specialist ______

____Medical Intuitive ______

128f. **Other** (please specify) Y/N

____ _____________________________ ______

_______________________________________ ______

_______________________________________ ______

1. After your emergency department visit, did you use any of the following treatments because of injuries or other health problems that were **related to your motor vehicle accident**?

Y/N Ice

Y/N Heat

Y/N Neck Immobilizers/Collars

Y/N Bed rest

Y/N Special Exercises/Yoga

Y/N Infrared

Y/N Other (please specify) ________________________________________________________________________

______________________________________________________________________________________________

1. After your motor vehicle accident, did you have to go back to the **emergency department** because of injuries or other health problems that were **related to your motor vehicle accident?** Y/N

**If yes**, what problems were you having? __________________________________________________________

___________________________________________________________________________________________

1. After your motor vehicle accident, **were you admitted to the hospital** because of injuries or other health problems that were **related to your motor vehicle accident?** Y/N

**If yes**, what were you admitted for? ______________________________________________________________

___________________________________________________________________________________________

1. After your motor vehicle accident, **did you have to have surgery** because of injuries or other health problems that were **related to your motor vehicle accident?** Y/N

**If yes**, what types of surgery did you receive? ______________________________________________________

___________________________________________________________________________________________

1. After your motor vehicle accident, **did you have to hire a nurse or someone else to help you at home** because of injuries or other health problems that were **related to your motor vehicle accident?** Y/N

**If yes**, who did you hire? ______________________________________________________________________

___________________________________________________________________________________________

1. After your motor vehicle accident, **have you had to have any other types of care not listed above** because of injuries or other health problems that were **related to your motor vehicle accident?** Y/N

**If yes**, what types of care did you receive? ________________________________________________________

___________________________________________________________________________________________

1. Do you currently have health insurance?

**0** ____ No

**1** ____ Yes

1. **If yes**, does your insurance cover doctor’s office visits and hospitalizations?

**0** ____ No

**1** ____ Yes, completely covered

**2** ____ Yes, partially covered (copay, or percentage)

1. **If yes**, does your insurance cover medications?

**0** ____ No

**1** ____ Yes, completely covered

**2** ____ Yes, partially covered (copay, or percentage)

1. Have you hired a lawyer, or are you working with a lawyer, because of injuries or other health problems that were **related to your motor vehicle accident**?

**0** ____ No

**1** ____ Yes

1. **If yes**, are you? (Select all that apply)

**1** ____Suing for damages or workman’s comp?

**2** ____Being sued?

**3** ____Other

Specify:

**Medications**

**The next questions are about medications that you take.**

140. In the **past month**, have you taken any over-the-counter pain medicines, such as Tylenol, Motrin, or Advil?

**0** ____ No

**1** ____ Yes

141. In the **past month**, did you take any prescription pain medicines? (Vicodin, Darvocet, or Tylenol #3, etc.)

**0** ____ No

**1** ____ Yes

Now I would like to make a list of all the medications that you currently take including the pain medications we just talked about. Please tell me all the medications you take even if you just take them once in a while. Include medicines that you take “over the counter” as well as medications that the doctor prescribes for you.

Route and Units Guide

| Routes | | Applicable Units |
| --- | --- | --- |
| Oral/PO | Liquids | micrograms (mcg) |
|  |  | milligrams (mg) |
|  |  | grams (g) |
|  |  | grains |
|  |  | teaspoons (tsp) |
|  |  | tablespoons (tbsp) |
|  |  | milliliters (ml) |
|  | Pills | micrograms (mcg) |
|  |  | milligrams (mg) |
|  |  | grams (g) |
|  |  | grains |
|  | Sub-lingual | sprays |
|  |  | tablets |
|  |  | milligrams |
| Injections (IV, Subcutaneous, IM) | | units |
|  |  | micrograms (mcg) |
|  |  | milligrams (mg) |
|  |  | ampules (amps) |
|  |  | milliliters (ml) |
| Inhaler | | puffs |
| Transcutaneous/Patch/Topical | | patches |
|  |  | vials |
|  |  | teaspoons (tsp) |
|  |  | inches |
| Eye/Ear Drop/Ointment | | drops (ggt) |
| Rectal | | suppository |
|  |  | tablet |
| Nasal/Throat Spray | | sprays |

| **Medications taken every day** | | | | | |
| --- | --- | --- | --- | --- | --- |
| Medication Name  (Include strength if any) | How do you take this?  (Pill, injection, liquid, inhaler, transcutanious/patch/topical, eye drop or ointment, ear drop, nasal spray, rectal) | How much do you take each time? (Include amount with unit) | How many times a day do you take?. | How long have you been taking this?  (days, weeks, months, years) | Reason for Taking? |
|  |  |  |  |  |  |
|  |  |  |  |  |  |
|  |  |  |  |  |  |
|  |  |  |  |  |  |
|  |  |  |  |  |  |
|  |  |  |  |  |  |
|  |  |  |  |  |  |
|  |  |  |  |  |  |
|  |  |  |  |  |  |
|  |  |  |  |  |  |
|  |  |  |  |  |  |
|  |  |  |  |  |  |
|  |  |  |  |  |  |
|  |  |  |  |  |  |
|  |  |  |  |  |  |
|  |  |  |  |  |  |

| **Medications Taken As Needed** | | | | | |
| --- | --- | --- | --- | --- | --- |
| Medication Name  (Include strength if any) | How do you take this?  (Pill, injection, liquid, inhaler, transcutanious/patch/topical, eye drop or ointment, ear drop, nasal spray, rectal) | How much do you take each time?  (Include amount with unit) | How often are do you take?  (per day, week month, year) | How long have you been taking this?  (days, weeks, months, years | Reason for Taking? |
|  |  |  |  |  |  |
|  |  |  |  |  |  |
|  |  |  |  |  |  |
|  |  |  |  |  |  |
|  |  |  |  |  |  |
|  |  |  |  |  |  |
|  |  |  |  |  |  |
|  |  |  |  |  |  |
|  |  |  |  |  |  |
|  |  |  |  |  |  |
|  |  |  |  |  |  |
|  |  |  |  |  |  |
|  |  |  |  |  |  |
|  |  |  |  |  |  |
|  |  |  |  |  |  |
|  |  |  |  |  |  |

142. Is there anything else that you would like to tell us? Is there anything else that we haven’t asked you, which is important for us to understand about the experience of being in a motor vehicle collision?

__________________________________________________________________________________________

______________________________________________________________________________________________________________________________________________________________________________________________________________________________________________________________________________

Before we end, we would like to verify that the contact information that we have listed for you is up to date. This information will help us reach you when it’s time for your follow-up interviews. The information you give us will be used only to locate you for your follow-up, and it will not be shared with anyone else outside of the Project CRASH Team. It will be destroyed after the conclusion of the study.

1. We have your current address as:

*CURRENT ADDRESS*

Is this your correct address? _____No ____ Yes

1. **If no**, what is your correct current address?

Street: __________________________________________________________________

City: _____________________________ State: _______ Zip: ________________

1. Do you plan on moving or changing your primary address between now and MONTH OF NEXT INTERVIEW?

_____No (GO TO 148)

_____Yes (GO TO 146)

1. On what date will you be moving? If you are not certain please give your closest estimate.

__/__/____

1. What will be you new street address.

Address_______________________________________________________

City: _____________________________ State: _______ Zip: _____________

Unknown? Y/N

1. Now we would like to confirm that we have the correct telephone information for you. Please check the following information to make sure that the phone numbers that we have listed for you are correct. Also, check that the best times to reach you are correct.

|  |  | Best times to reach you: | | | ***Skip these questions if you will not be moving in the next two months.*** | |
| --- | --- | --- | --- | --- | --- | --- |
| TYPE | PHONE | Day of the week | Start Time | End Time | Will your upcoming move change this number? | If yes, and you know it, What will your new number be? |
| Home | XXX-XXX-XXXX |  | _ _ : _ _ | _ _ : _ _ | Yes / No | XXX-XXX-XXXX |
|  |  |  | _ _ : _ _ | _ _ : _ _ |  |  |
|  |  |  | _ _ : _ _ | _ _ : _ _ |  |  |
| Cell | XXX-XXX-XXXX |  | _ _ : _ _ | _ _ : _ _ | Yes / No | XXX-XXX-XXXX |
|  |  |  | _ _ : _ _ | _ _ : _ _ |  |  |
|  |  |  | _ _ : _ _ | _ _ : _ _ |  |  |
| Work/Other | XXX-XXX-XXXX |  | _ _ : _ _ | _ _ : _ _ | Yes / No | XXX-XXX-XXXX |
|  |  |  | _ _ : _ _ | _ _ : _ _ |  |  |
|  |  |  | _ _ : _ _ | _ _ : _ _ |  |  |

**Updated information**

If any of the information above is incorrect please fill in the correct information in this chart.

|  |  | Best times to reach you: | | |
| --- | --- | --- | --- | --- |
| TYPE | PHONE | Day of the week | Start Time | End Time |
| Home | XXX-XXX-XXXX |  | _ _ : _ _ | _ _ :_ _ |
|  |  |  | _ _ : _ _ | _ _ : _ _ |
|  |  |  | _ _ : _ _ | _ _ : _ _ |
| Cell | XXX-XXX-XXXX |  | _ _ : _ _ | _ _ : _ _ |
|  |  |  | _ _ : _ _ | _ _ : _ _ |
|  |  |  | _ _ : _ _ | _ _ : _ _ |
| Work/Other | XXX-XXX-XXXX |  | _ _ : _ _ | _ _ : _ _ |
|  |  |  | _ _ : _ _ | _ _ : _ _ |
|  |  |  | _ _ :_ _ | _ _ :_ _ |

1. Which number would you like for us to try and reach you at first? (Check one)

Home ___

Cell ___

Work or Other ___

1. You listed the following contact information for a person who could give us your new address in case we can’t get in touch with you.

*CONTACT INFO #1*

Is this information still correct, and may we still contact this person to get your new address?

_____No ____ Yes

- 1. **If no**, could you provide us with the correct information or the information of another person who you do not currently live with that could give us your new address? We would contact this person only if we are unable to reach you using other information that you have given us.


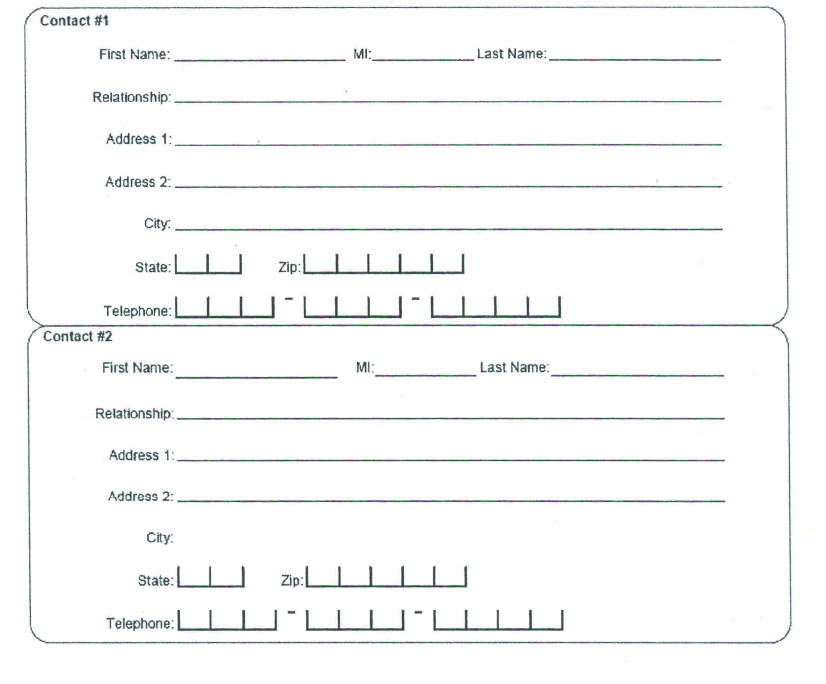


1. You also listed the following contact information for a second person who could give us your new address in case we can’t get in touch with you.

*CONTACT INFO #2*

Is this information still correct, and may we still contact this person to get your new address?

_____No ____ Yes

- 1. **If no**, could you provide us with the correct information or the information of a second person who does not currently live with you that could give us your new address? We would contact this person only if we are unable to reach you using other information that you have given us.


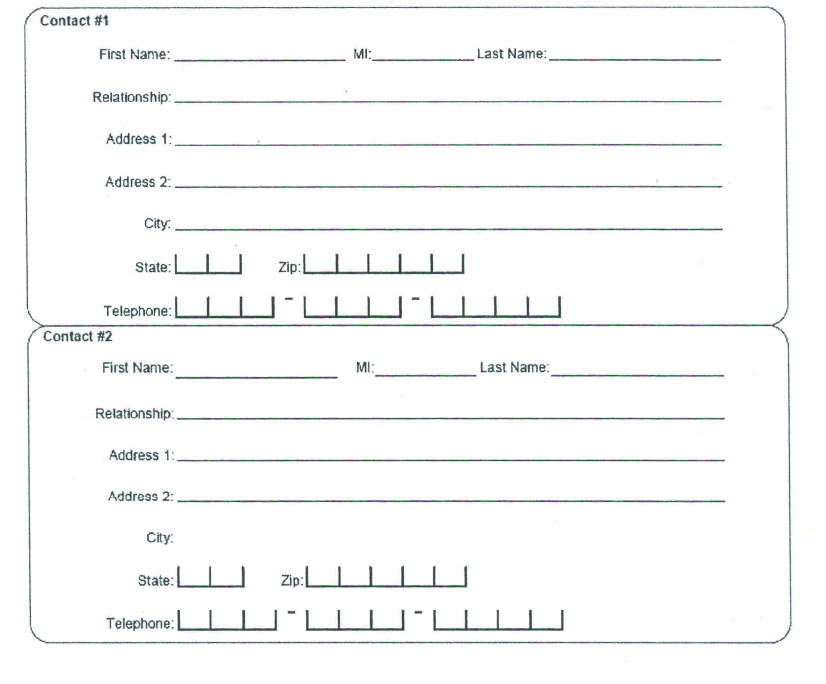


1. You may have provided us with your email addresses. If so, are the following addresses still correct:

*EMAIL#1 _____No ____ Yes*

*EMAIL#2 _____No ____ Yes*

*EMAIL#3 _____No ____ Yes*

1. Please list your corrected email addresses if any of the above are incorrect or any email address(s) that you check regularly that are not listed above:

#1 ___________________________________________________

#2 ___________________________________________________

#3 ___________________________________________________
